# De novo selected hACE2 mimics that integrate hotspot peptides with aptameric scaffolds for binding tolerance of SARS-CoV-2 variants

**DOI:** 10.1126/sciadv.abq6207

**Published:** 2022-10-26

**Authors:** Minjong Lee, Byunghwa Kang, Juhwa Lee, Jisun Lee, Sang Taek Jung, Chang Yun Son, Seung Soo Oh

**Affiliations:** ^1^ Department of Materials Science and Engineering, Pohang University of Science and Technology (POSTECH), Pohang 37673, South Korea.; ^2^ Department of Chemistry, Pohang University of Science and Technology (POSTECH), Pohang 37673, South Korea.; ^3^ Department of Biomedical Sciences, Graduate School, Korea University, Seoul 02841, South Korea.; ^4^ BK21 Graduate Program, Department of Biomedical Sciences, Korea University College of Medicine, Seoul 02841, South Korea.; ^5^ Institute for Convergence Research and Education in Advanced Technology (I-CREATE), Yonsei University, Incheon 21983, South Korea.

## Abstract

The frequent occurrence of viral variants is a critical problem in developing antiviral prophylaxis and therapy; along with stronger recognition of host cell receptors, the variants evade the immune system–based vaccines and neutralizing agents more easily. In this work, we focus on enhanced receptor binding of viral variants and demonstrate generation of receptor-mimicking synthetic reagents, capable of strongly interacting with viruses and their variants. The hotspot interaction of viruses with receptor-derived short peptides is maximized by aptamer-like scaffolds, the compact and stable architectures of which can be in vitro selected from a myriad of the hotspot peptide-coupled random nucleic acids. We successfully created the human angiotensin-converting enzyme 2 (hACE2) receptor–mimicking hybrid ligand that recruits the hACE2-derived receptor binding domain–interacting peptide to directly interact with a binding hotspot of severe acute respiratory syndrome coronavirus 2 (SARS-CoV-2). Experiencing affinity boosting by ~500% to Omicron, the de novo selected hACE2 mimic exhibited a great binding tolerance to all SARS-CoV-2 variants of concern.

## Teaser

Hotspot peptide-aptamer conjugates recognize all kinds of viral variants that have evolved for stronger receptor binding.

## INTRODUCTION

For infection, many viruses specifically recognize cellular membrane proteins during their entry into host cells (*1*). For example, severe acute respiratory syndrome coronavirus 2 (SARS-CoV-2), as covered by thousands of spike proteins, strongly bind to human angiotensin-converting enzyme 2 (hACE2), a membrane receptor, leading to its internalization by membrane fusion (*2*, *3*). To be more infectious, viruses including SARS-CoV-2 often mutate, enhancing viral transmission and immune escape over time (*4*, *5*). Since the first report of SARS-CoV-2 in 2019, many variants have emerged to date, and the five variants of concern (VOCs)—Alpha (B.1.1.7), Beta (B.1.351), Gamma (P.1), Delta (B.1.617.2), and Omicron (B.1.1.529)—have independently occurred on different continents (*6*). The receptor binding domain (RBD) of the spike protein, which is attributed to the specific hACE2 recognition, has been known to include a lot of common mutations (*5*); in particular, some site mutations (e.g., N501Y), located in the binding hotspots, were identified to increase the binding affinity of the RBD to the targeted hACE2, thereby elevating the transmissibility of SARS-CoV-2 (*7*, *8*).

The frequent occurrence of variants can be a substantial problem in developing antiviral prophylaxis and therapy. To avoid viral infection, use of affinity reagents can be an effective way by blocking specific interactions between viruses and host cells; against SARS-CoV-2, a number of neutralizing affinity reagents have been developed to recognize various epitopes on the spike protein (*9*–*12*), some of which are known to partly overlap the hACE2-contacting surface. However, as escape mutations cause the structural change of the spike proteins, affinity reagents are not allowed for the specific recognition of their binding sites, thereby leading to an inevitable decrease in neutralizing efficacy (*5*, *13*–*16*). As alternatives, noncompeting affinity reagents can be administered together, and the U.S. Food and Drug Administration (FDA) issued the emergency use authorization of antibody cocktails, such as REGN-COV2, because of the rapid emergence of SARS-CoV-2 variants (*17*, *18*). By accumulating escape mutations, however, viral variants tend to become more resistant to the mixture of potent antibodies, although their binding interaction with host cell receptors is further strengthened (*19*). Along with the high affinity to target viruses, the binding tolerance to their variants should not be ignored in developing effective and efficient neutralizers to fight with continually evolving, life-threatening viruses (*20*–*22*).

Inspired by the enhanced receptor recognition of viral variants, we newly demonstrate the generation of receptor-mimicking synthetic reagents capable of strongly interacting with target viruses and even their variants. We focused on a peptide motif of a host cell receptor, which heavily contributes to the binding free energy at the center of the virus-receptor interface. Without a stable yet insoluble transmembrane domain, the short hotspot peptide cannot maintain its optimal binding ability to the target virus (*23*–*27*); in this work, it was synergistically integrated with exceptionally soluble nucleic acids that can serve not only as a structural stabilizer but also as a binding cooperator. From a myriad of hotspot peptide-coupled random nucleic acids (~10^14^), we can readily discover the hybrid ligands by selectively isolating and amplifying aptamer-like scaffolds that maximize the hotspot interaction, which can lead to the invariable strong binding to viral variants. Using our novel in vitro selection technique, termed “hotspot-oriented ligand display (HOLD),” we successfully created the hACE2 receptor–mimicking hybrid ligand that recruits the hACE2-derived RBD-binding peptide to directly interact with a binding hotspot of SARS-CoV-2. The synergistic interplay between the hotspot peptide and the aptameric scaffold achieved comparable or more competitive binding to the RBD than the reported affinity reagents (e.g., cyclic peptides, aptamers, or neutralizing antibodies), assuring efficient blocking of SARS-CoV-2. In recognizing various SARS-CoV-2 variants, our hACE2 mimic of hotspot binding did not alter its binding ability, and unexpectedly, its original equilibrium dissociation constant (*K*_d_ = 5.702 nM) decreased approximately by five times, thereby reserving the great binding tolerance toward all the representative VOCs: Alpha, Beta, Gamma, Delta, and even the recently reported Omicron.

## RESULTS

### Receptor-mimicking hybrid ligand and its hotspot-oriented in vitro selection

**Fig. 1.**
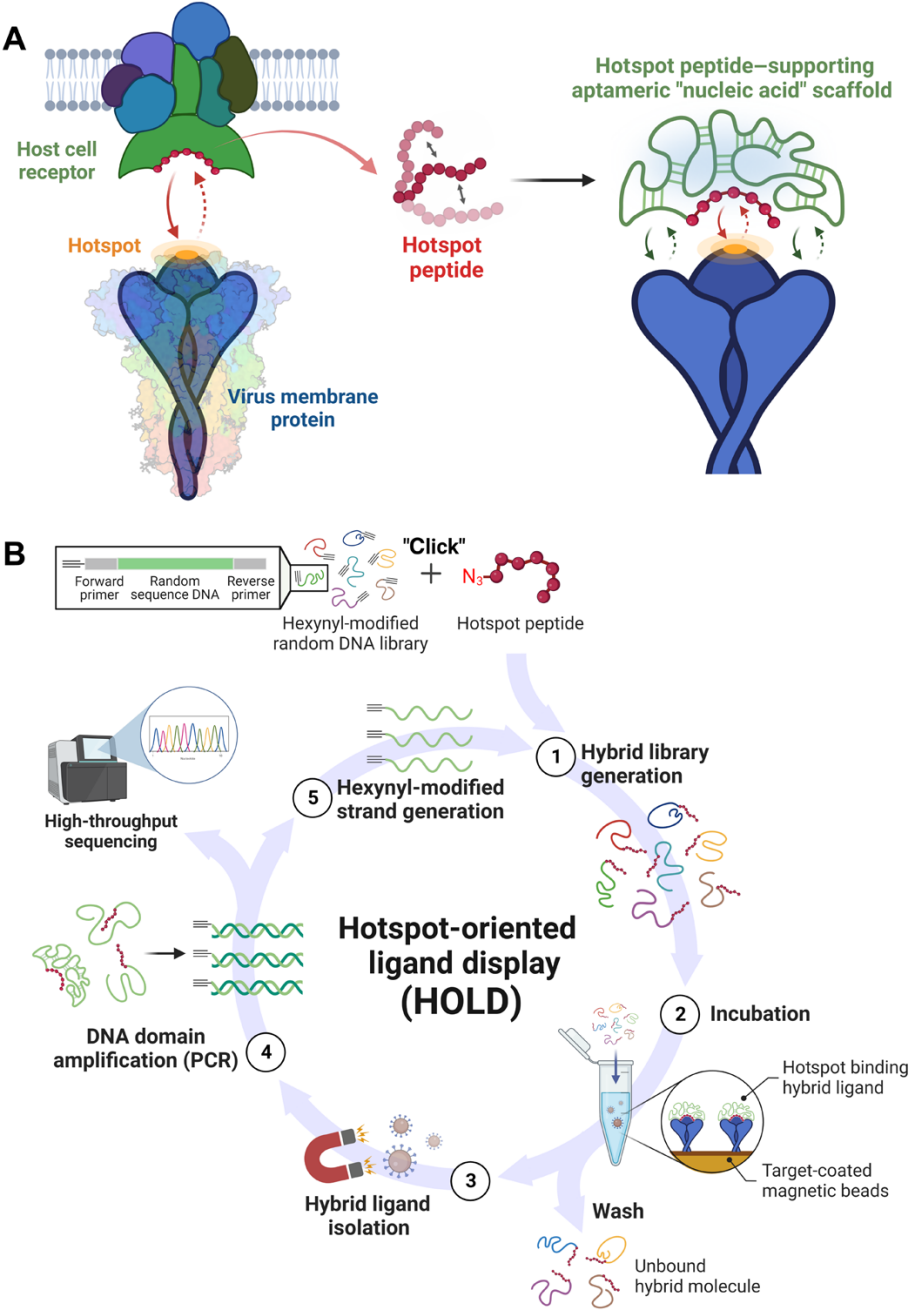
In vitro selection of receptor-mimicking hybrid ligand composed of a hotspot peptide and an aptamer-like nucleic acid scaffold. (**A**) Hotspot peptide-supporting aptameric scaffold as a receptor mimic. Even after deriving from the binding hotspot of host cell receptors, the flexible hotspot peptide enables the strong interaction with the viral membrane protein in a right position and orientation as synergistically supported by the compact and hydrophilic nucleic acid scaffold. (**B**) Overview of HOLD. (1) The starting hybrid random library (~10^14^ molecules) is prepared by the efficient click reaction that assures site-specific conjugation between an azide-tagged hotspot peptide with a hexynyl-modified single-stranded DNA (ssDNA) library. (2) When incubated with target-coated magnetic beads, hotspot binding hybrid ligands are readily accommodated on the bead surface, allowing the unbound hybrid molecules to be washed away. (3) Use of magnet enables the selective isolation of the hybrid ligand candidates, (4) of which DNA domains are subsequently amplified by the polymerase chain reaction (PCR) in the presence of 5′-hexynyl–modified forward primers. (5) After single-strand generation, the hexynyl-modified ssDNAs are ready to be coupled with the azide-tagged hotspot peptides for the next round of the HOLD process.

By appropriate folding, nucleic acids can provide highly stable and water-soluble three-dimensional (3D) scaffolds that can place hotspot peptides in a right position and orientation to maintain their receptor-like binding properties (Fig. 1A). For the specific RBD-hACE2 interaction, several hotspot peptides have been reported to be roughly 30 amino acids or less in length (*28*–*30*), but while apart from the insoluble hACE2 transmembrane receptor, the flexible motifs cannot be strongly bound to the hotspot surface of the RBD (*23*). It is well known that, along with negligible immunogenicity, the nucleic acids are exceptionally hydrophilic because of the polar nature of their phosphate backbones. Moreover, systematically designed iterative cycles of selection and amplification can lead to in vitro selection of unique nucleic acids (*31*), capable of structurally stabilizing the hACE2-derived RBD-binding peptides. Without massive and insoluble domains, the compact and highly soluble peptide stabilizers would permit high-dose intravenous administration for viral neutralization (*32*, *33*). Moreover, the role of the nucleic acids can be further extended as a hotspot binding enhancer by cooperatively interacting with the target RBD (*34*, *35*); centered on the monovalent peptide binding, there can be additional aptamer-like interactions, further improving the hotspot binding ability of the hACE2-mimicking hybrid ligand.

From a myriad of different nucleic acids (~10^14^) that are site-specifically linked with hotspot peptides, the peptide-supporting aptameric scaffolds can be in vitro isolated by iterative cycles of selection and amplification (Fig. 1B). Initially, the azide-tagged C terminus of peptides can be site-specifically conjugated to the 5′-hexynyl end of single-stranded DNA (ssDNA) library by highly efficient copper-catalyzed cycloaddition (Fig. 1B, step 1, and fig. S1), and after purification and thermal denaturation, the peptide-linked random DNA library is snap-cooled for unique 3D folding of individual ssDNAs. Upon introduction of RBD-coated magnetic beads, the RBD surfaces attract the hotspot interaction of hybrid ligand candidates, and magnetic separation allows unbound peptide-DNA conjugates to be systematically retarded (Fig. 1B, steps 2 and 3). Subsequently, the DNA domain of the bead-bound hybrid ligands can be selectively amplified by polymerase chain reaction (PCR) with 5′-hexynyl–modified forward primers and 5′-phosphorylated reverse primers (Fig. 1B, step 4, and fig. S2A). Using lambda exonuclease digestion, we can generate 5′-hexynyl–modified ssDNAs (fig. S2B), of which click reaction with the azide-tagged hotspot peptides provides the same format of hybrid library pool again for the next round of the evolutionary HOLD process (Fig. 1B, step 5).

### SARS-CoV-2 RBD-binding hotspot peptide and hACE2 mimic generation

Among RBD-contacting residues of hACE2, a seven–amino acid fragment from L351 to R357 was chosen to be fused with the random DNA library because of its strong hotspot interaction with the RBD of SARS-CoV-2 (*30*, *36*). Within the short and consecutive chain, four amino acids (K353, G354, D355, and R357) have been confirmed to directly contact the surface of the RBD by cryo–electron microscopy (cryo-EM) observation (fig. S3) (*2*, *36*). This hotspot region is relevant to the binding affinity of SARS-CoV-2 VOCs; because of the N501Y mutation of the RBD, the VOCs bind more tightly and strongly to hACE2 because the K353 of hACE2, the central amino acid of the chosen hotspot peptide, is involved in an unexpected hydrophobic interaction with the substituted tyrosine (fig. S4) (*7*, *8*). Moreover, this LGKGDFR peptide includes two hydrophobic and two positively charged amino acids, the nature of which cannot be offered by fully hydrophilic and negatively charged DNAs; thus, the hotspot peptide-linked random DNA library is expected to cover highly diverse electrostatic interactions in evolving the highest affinity to the RBD of SARS-CoV-2 (*35*, *37*, *38*).

**Fig. 2.**
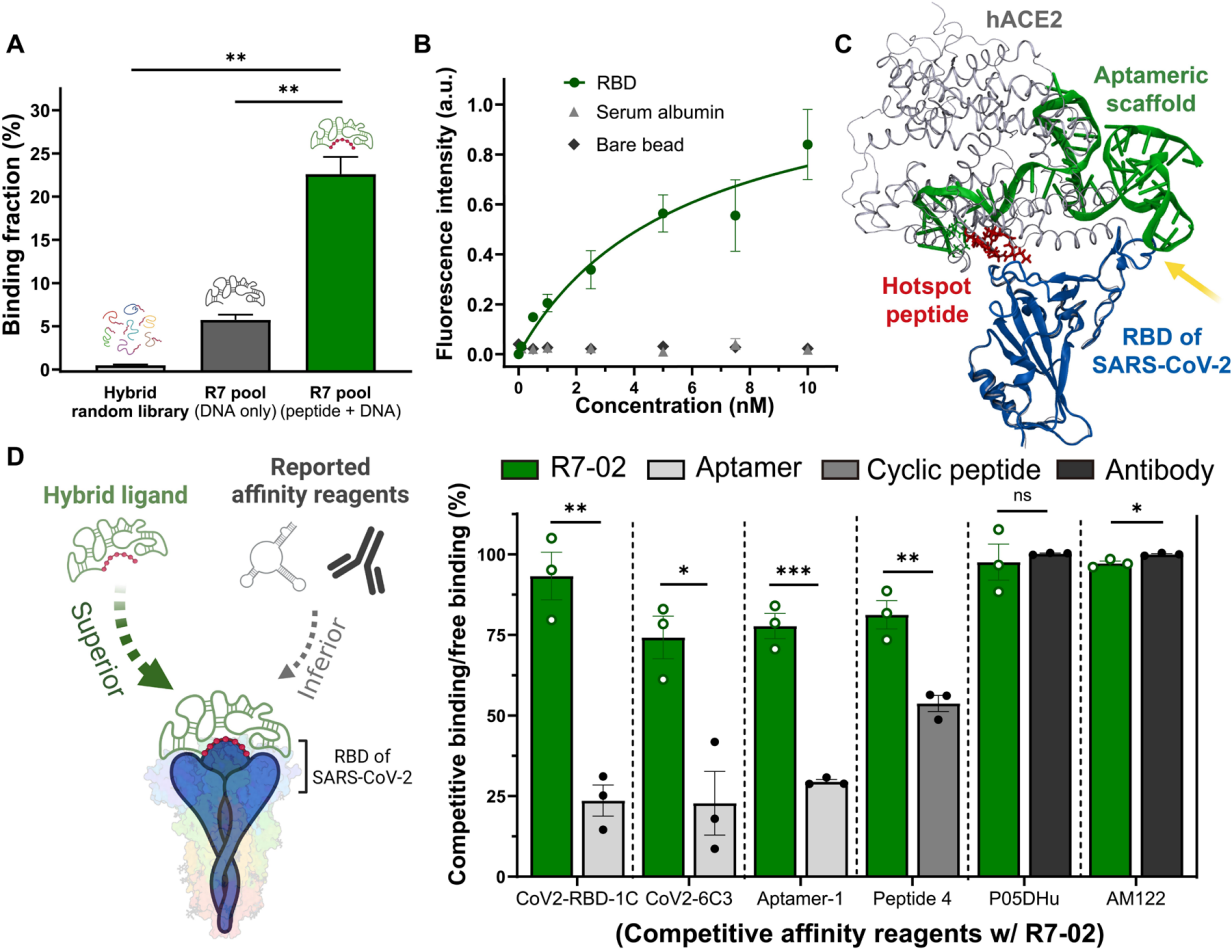
Generation of hACE2-mimicking hybrid ligand with high affinity and specificity to the RBD of SARS-CoV-2. (**A**) Synergistic interplay between the hotspot peptide and the newly evolved aptameric scaffold pool. The RBD-binding fraction was compared among three different groups: the random DNA library coupled with the hotspot peptide (light gray), the R7 ssDNA pool without the hotspot peptide (dark gray), and the hotspot peptide-supporting R7 ssDNA pool (green). (**B**) Affinity and specificity characterization of R7-02 as the strongest RBD binder. The RBD binding–dependent fluorescence intensity was plotted in varying concentrations of R7-02 (0 to 10 nM) for calculation of *K*_d_. When incubated with three different types of beads, RBD- and serum albumin–coated beads (green and gray, respectively) and bare beads (black), R7-02 exhibited a *K*_d_ of 5.702 nM for the RBD, but its negligible binding was observed to the serum albumin and the bare beads. Fifteen thousand beads were analyzed for each measurement. *n* = 3 independent replicates (bars represent means ± SD). (**C**) Energy-minimized structure of R7-02, bound to a single RBD, obtained by molecular docking simulation. The RBD, hotspot peptide, and aptameric DNA scaffold of R7-02 are in blue, red, and green, respectively. The crystal structure of the RBD-bound hACE2 protein (6M0J) is represented in white. (**D**) Competitive binding analysis with reported RBD-binding affinity reagents. When our hybrid ligand exclusively covers its binding epitope on the RBD, the other affinity reagents in competition cannot be accommodated on the RBD surface (left). The RBD-binding amount of the hybrid ligand was measured with and without its competitors to calculate the ratio of competitive binding to free binding (green), and the ratios for the other affinity reagents (light gray, gray, and black for aptamers, cyclic peptide, and antibodies, respectively) were obtained in a similar way. *n* = 3 independent replicates (*P < 0.05, **P < 0.01, and ***P < 0.001; bars represent means ± SEM). ns, not significant.

From seven rounds of in vitro selection with gradually increased stringency (table S1), a hybrid ligand pool was successfully enriched to have a strong binding affinity to the RBD because of the synergistic effect between the hotspot peptide and the aptameric scaffold (Fig. 2A and figs. S5 and S6). From a relative binding assay, we quantitatively evaluated the RBD binding of the hotspot peptide-linked round 7 (R7) pool (see Materials and Methods and fig. S6). Despite the linkage of the hotspot peptide, the random DNA library displayed a significantly low level of RBD binding; when the peptide-linked DNA library was challenged with the RBD-coated magnetic beads, its binding fraction was only 0.38%, indicating that the hotspot peptide could not strongly bind to the RBD without a stable scaffold. In the absence of the LGKGDFR peptide, the R7 pool slightly increased RBD binding, exhibiting 5.74% binding fraction, presumably because of its own aptameric binding effect. In contrast, as the peptide-supporting R7 pool markedly improved its affinity to the RBD, its binding fraction (22.6%) became 59.37 and 3.94 times larger than that of the random DNA-peptide conjugates and the peptide-lacking R7 pool, respectively. Such a remarkable improvement in RBD binding would be attributed to the synergistic interplay between the hotspot peptide and the newly evolved aptameric scaffold.

### Identification and characterization of peptide-supporting aptameric scaffold

The most optimal peptide-supporting aptameric scaffold was identified from the R7 hybrid ligand pool using high-throughput sequencing and flow cytometry–based binding assay (Fig. 2B and fig. S7). When more than 4 million DNAs were sequenced from the R7 pool, the three most abundant sequence families were revealed to have high occupancy (>18%) (fig. S7A). Among the three sequence families, the most representative DNA sequences were synthesized with the 5′-hexynyl group for the site-specific peptide conjugation and 3′-fluorescein amidite (FAM) dye for a flow cytometry–based binding analysis (see Materials and Methods). With the 5′-linked hotspot peptide, the second most abundant DNA sequence (R7-02) was the strongest in RBD binding; in the relative binding assay, the RBD-binding fraction of R7-02 was approximately five and eight times larger than that of R7-01 and R7-03, respectively (fig. S7B). We note that the most enriched aptamers by in vitro selection have often exhibited inferior binding ability to targets, presumably because of several plausible biases in enrichment, such as the biased amplification of PCR-favorable DNAs (*39*).

To serve as a hACE2 mimic, the strongest hybrid ligand, R7-02, exhibited high affinity and specificity to the RBD at the right position where hACE2 can be bound. When we quantitatively measured the *K*_d_ of R7-02, it was 5.702 nM for the RBD (Fig. 2B, green), along with negligible binding to nontargets: carboxylic magnetic beads used for the in vitro selection (black) and serum albumin as the most abundant blood protein (gray); in agreement with the relative binding assay, the *K*_d_ values of R7-01 and R7-03 were much higher than that of R7-02 (fig. S7C). From molecular docking simulation (Fig. 2C), we predicted that the high binding affinity of R7-02 to the RBD would be attributed to the cooperative binding effect. In the energy-minimized simulation snapshot, the LGKGDFR peptide (red) is precisely positioned at the binding hotspot of original hACE2 (white) with the RBD (blue), and its binding is effectively stabilized with the conjugated aptameric scaffold (green). The DNA folding at the 3′ end provides an additional binding motif to the loop structure (A475-N487) of the RBD (yellow arrow), leading to the strong and specific RBD binding of R7-02; the exact positioning of R7-02 at the interface between the RBD and hACE2 proves the potential for SARS-CoV-2 neutralization at low nanomolar concentrations in physiological body fluids.

Even in competition with previously reported RBD-binding affinity reagents, R7-02 ensured highly sustainable binding retention to the targeted surface of RBDs (Fig. 2D). To induce the RBD-binding competition, our hybrid ligand of the hotspot peptide and the aptameric scaffold was co-incubated with RBD-binding nucleic acid aptamers [CoV2-RBD-1C (*40*), CoV2-6C3 (*41*), and Aptamer-1 (*12*)], macrocyclic peptides [Peptide 4 (*42*)], or monoclonal antibodies (P05DHu and AM122), most of which are known to block the binding interface between the RBD and hACE2 (Fig. 2D, left), and for fair comparison, the *K*_d_ values of the various RBD binders were determined under the same condition for R7-02 (fig. S8). In evaluating the competitive binding (see Materials and Methods), we measured the RBD-binding fraction of R7-02 with and without its potential binding competitors, and the decrease in RBD binding was quantitatively compared with that of the other affinity reagents (Fig. 2D, right). Even after 1 hour of co-incubation with the RBD-binding aptamers or cyclic peptides, R7-02 could maintain its RBD binding, exhibiting low levels of decrease (down to 6.8%) in a ratio of competitive binding to free binding. On the other hand, the RBD binding of all other aptamers and the cyclic peptides was significantly suppressed, with up to 77.2% decrease of the ratio between competitive and free binding, indicating that the binding of the monomeric aptamers and the cyclic peptides would be relatively weak to occupy the restrictive RBD binding site against the competition with our cooperative hybrid ligand. Presumably, because of the different binding sites, negligible competition was observed between the R7-02 and the P05DHu and AM122 antibodies.

### Binding tolerance of hACE2 mimic to SARS-CoV-2 VOCs

**Fig. 3.**
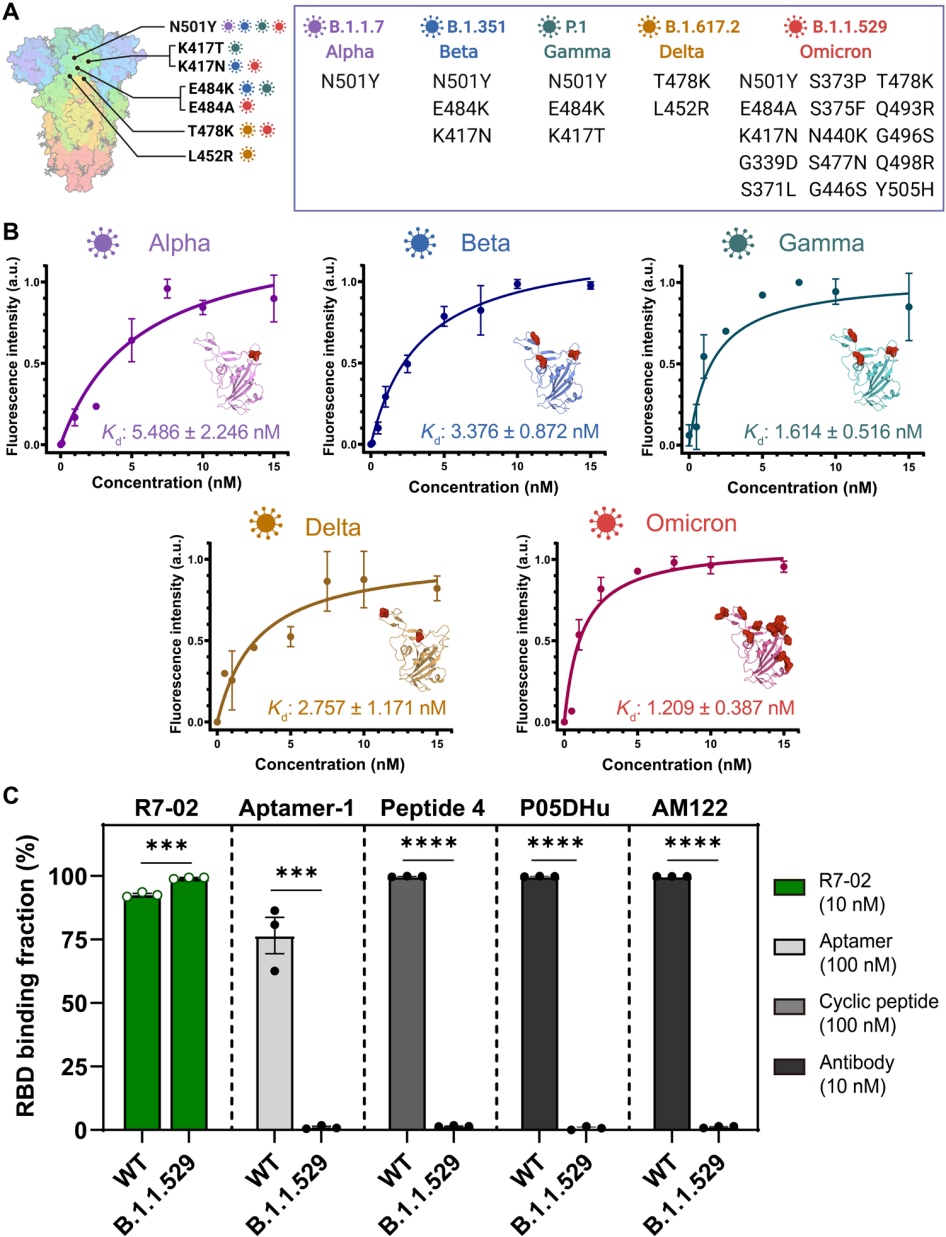
Binding tolerance of hACE2 mimic to all reported VOCs. (**A**) Schematic and lists of RBD mutations for the five reported VOCs: Alpha, Beta, Gamma, Delta, and Omicron. (**B**) Affinity characterization of R7-02 for the five different VOCs. The mutated RBD binding–dependent fluorescence intensity was plotted in varying concentrations of R7-02 (0 to 15 nM) to calculate *K*_d_ values for all the VOCs. Fifteen thousand beads were analyzed for each measurement. *n* = 3 independent replicates (bars represent means ± SD). The mutated residues in the RBD for every VOC are structurally represented by red spheres (insets for each graph). (**C**) Omicron RBD binding of R7-02 and the reported affinity reagents. The flow cytometry–based RBD binding fractions for wild type (WT) and Omicron (B.1.1.529) were plotted for each affinity reagent. *n* = 3 independent replicates (***P < 0.001 and ****P < 0.0001; bars represent means ± SEM).

With the great variant tolerance in binding, our hotspot peptide-supporting aptameric scaffold was confirmed to strongly bind all the reported VOCs of SARS-CoV-2 (Fig. 3). Although it was initially evolved to bind the wild-type RBD through our HOLD process, the binding ability of the hybrid ligand was not undermined to recognize the highly mutated RBDs of the VOCs (Fig. 3A), as attributed to the systemic architecture that strongly interacts with the hotspot interface of the RBD. The VOCs have been revealed to improve their binding to hACE2 receptors and transmissibility into host cells by having several key mutations, including N501Y, which is highly interactive with the lysine of the LGKGDFR peptide in the hybrid ligand. When we quantitatively evaluated the binding of R7-02 to the five different VOCs, the *K*_d_ values of R7-02 (5.486, 3.376, 1.614, 2.757, and 1.209 nM for Alpha, Beta, Gamma, Delta, and Omicron, respectively) were unexpectedly lower than that for the wild-type RBD (5.702 nM), validating the binding tolerance of R7-02 to all the variants (Fig. 3B). Omicron, having the most abundant RBD mutations, was the most strongly recognized by R7-02, while many different types of chosen RBD binders (Aptamer-1, Peptide 4, P05DHu, and AM122) lost their RBD binding abilities owing to the heavy mutations (Fig. 3C and fig. S9). This observation confirmed that the binding tolerance of R7-02 to the SARS-CoV-2 VOCs would be unique, and it is supposed that the hotspot peptide-oriented RBD binding could be significantly influenced by the mechanism of the improved hACE2 recognition by SARS-COV-2 VOCs.

### Inhibition of RBD-hACE2 interaction and neutralization of pseudotyped SARS-CoV-2

**Fig. 4.**
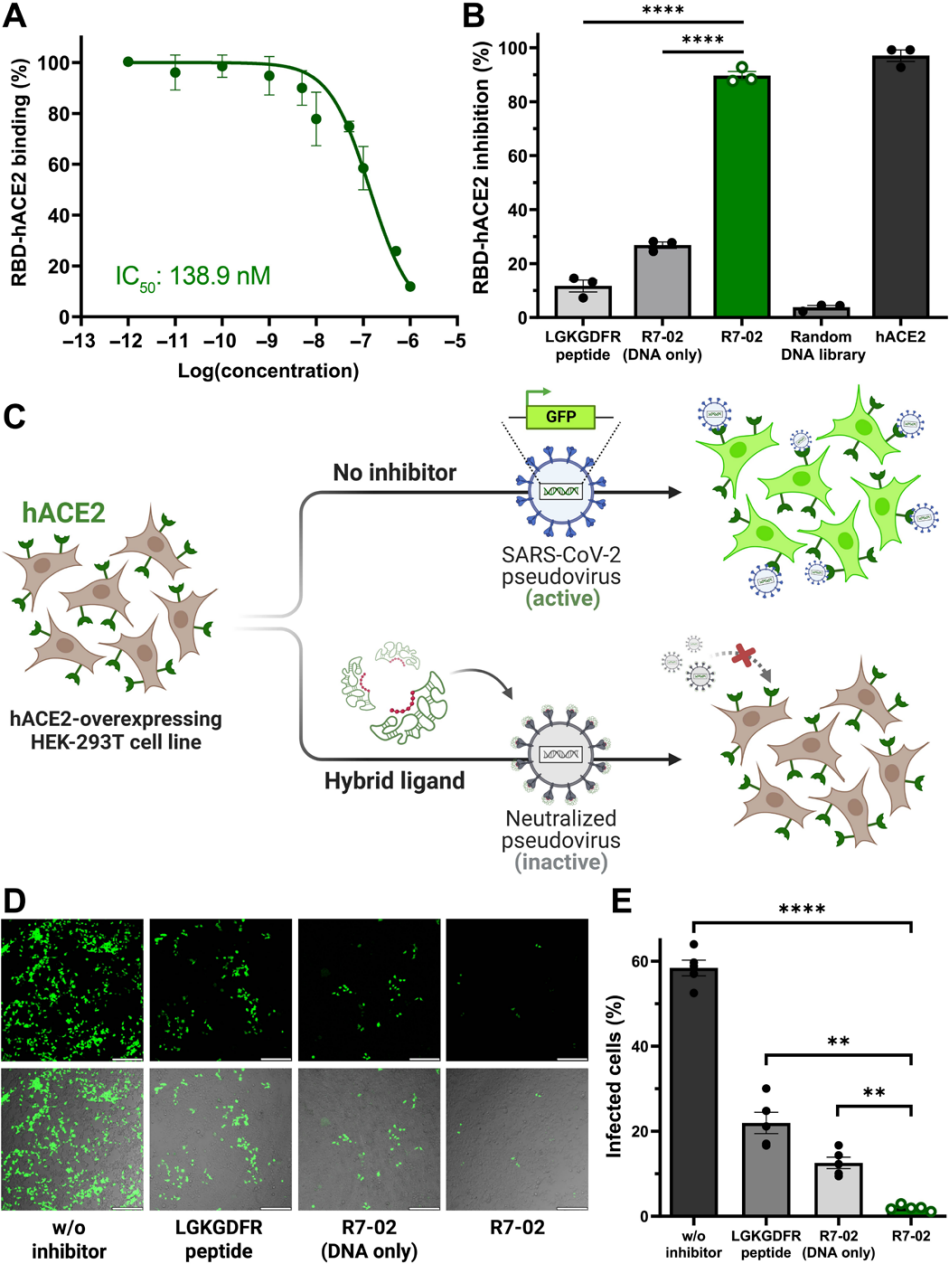
Efficient RBD-hACE2 inhibition and pseudotyped SARS-CoV-2 neutralization. (**A**) ELISA-based RBD-hACE2 binding inhibition test. When the percentage of the RBD-hACE2 binding was plotted in varying concentrations of R7-02 (1 pM to 1 μM), the IC_50_ value for the R7-02–driven inhibition of the RBD-hACE2 binding was determined to be 138.9 nM. (**B**) Confirmation of synergistic interplay between the hotspot peptide and the aptameric scaffold in inhibiting the RBD-hACE2 interaction. The percentage of the RBD-hACE2 inhibition was measured by ELISA after 3 μM treatment of individual components. *n* = 3 independent replicates. (**C**) Schematic of pseudotyped SARS-CoV-2–based neutralization assay. While carrying the gene of wild-type SARS-CoV-2 spike protein and green fluorescence protein (GFP), the pseudotyped SARS-CoV-2 can infect hACE2-overexpressing human embryonic kidney (HEK) 293T cell lines (hACE2-293T) to cause GFP overexpression emitting strong green fluorescence (top). In contrast, the successful inhibition of RBD-hACE2 binding can lead to no fluorescence emission due to no infection of the pseudotyped SARS-CoV-2 (bottom). (**D**) Confocal microscopic observation of pseudotyped SARS-CoV-2–infected hACE2-293T. The green fluorescence images (top) and the merged images of the green fluorescence with the bright field (bottom) were observed with or without inhibitors. Scale bars, 200 μm. Excitation: 480 nm and emission: 530 nm. (**E**) Percentage of the GFP-expressing infected cells over the total hACE2-293T cells. Around 1000 to 2000 cells were analyzed for each sample. *n* = 5 biological replicates (**P < 0.01 and ****P < 0.0001; bars represent means ± SEM).

Because of the strong binding to the RBD, the hotspot peptide-supporting aptameric scaffold efficiently inhibited the interaction between the RBD of SARS-CoV-2 and the hACE2 receptor even at nanomolar concentrations. For characterization of inhibition efficiency, we performed an enzyme-linked immunosorbent assay (ELISA) while varying the concentrations of R7-02 from 1 pM to 1 μM (see Materials and Methods), and R7-02 exhibited a half-maximal inhibitory concentration (IC_50_) of 138.9 nM for the RBD-hACE2 binding inhibition (Fig. 4A). The RBD inhibition of R7-02 was guided by the synergistic effect of the systematically combined hotspot peptide and the in vitro evolved aptameric scaffold; the individual components of R7-02, the LGKGDFR peptide and the aptameric scaffold, only showed 11.69 and 26.82% inhibition of RBD-hACE2 interaction at their concentrations of 3 μM, respectively, whereas the full structure of R7-02 succeeded in 89.60% inhibition. Compared to the random DNA library (3.77% in inhibition), the DNA scaffold alone exhibited a better inhibition efficiency, as evidence of its aptameric behavior (Fig. 4B). Previously, several RBD-binding affinity reagents failed to effectively inhibit the RBD-hACE2 interaction because they could not block the exact hACE2-contacting residues on the RBD surface (*43*). In contrast, the hotspot-oriented RBD binding of R7-02 proved the efficient inhibition of the RBD in hACE2 binding, posing a great potential for SARS-CoV-2 neutralization.

Encouraged by superior RBD binding and blocking, the hotspot peptide-supporting aptameric scaffold was further investigated with respect to the ability of SARS-CoV-2 neutralization. For the neutralization assay, we prepared the pseudotyped SARS-CoV-2, expressing both SARS-CoV-2 spike proteins and green fluorescent proteins (GFPs), but lacking the function of self-replication (*44*); by measuring the fluorescence intensity of GFPs, which are only expressed in infected cells by the pseudotyped SARS-CoV-2, the neutralizing efficiency of R7-02 could be analyzed fluorescently (Fig. 4C). Briefly, hACE2-overexpressing human embryonic kidney (HEK) 293T cell lines (hACE2-293T) were preseeded into microplates overnight and subsequently challenged with the pseudotyped SARS-CoV-2 (0.5 multiplicity of infection) for 6 hours for viral infection (see Materials and Methods). With no RBD inhibitors, hACE2-293T was well infected by the pseudotyped SARS-CoV-2, and quantitatively, 58.4% of the total cells emitted strong green fluorescence as evidence of successful viral infection (Fig. 4, D and E). In the presence of either hotspot peptides or aptameric scaffolds comprising R7-02, the infection of the pseudotyped SARS-CoV-2 was diminished, presumably because of a limited level of their RBD binding, causing their infection rates to be down to 22.0 and 12.6%, respectively. Notably, R7-02, the hotspot peptide-supporting aptameric scaffold, most efficiently hindered the infection of hACE2-293T with the pseudotyped SARS-CoV-2, as evidenced by negligible green fluorescence (Fig. 4D) and a low percentage (1.9%) of infected cells (Fig. 4E). Even when compared to viral neutralization of many different RBD binders, that of R7-02 was revealed to be superior or comparable (fig. S10), and it should be considered that, unlike the most commonly used neutralizing antibodies, our hybrid ligands are fully synthetic and have a low molecular weight (20 kDa), which is about 110 of that of monoclonal antibodies, but they ensure efficient infection inhibition. Furthermore, with no further modification, the chimeric structure of the peptide-aptamer hybrid ligand displayed decent nuclease resistance and serum stability, comparable to those of well-known oligonucleotide modifications (e.g., phosphorothioate backbone, and 2′-*O*-methyl and 2′-F modifications) (fig. S12), posing a great potential for SARS-CoV-2 neutralizers.

## DISCUSSION

In this study, we demonstrate the novel HOLD technique capable of generating hybrid ligands that synergistically integrate receptor-derived hotspot peptides with cooperatively evolved aptameric scaffolds. Original transmembrane receptors have critical drawbacks as viral neutralizers; the lipophilic membrane proteins are insoluble and could not be readily administered in our bodies (*11*), and their massive size can cause steric hindrance in covering the surface of viruses, such as coronaviruses with high-density surficial spike proteins (*45*). On the other hand, highly soluble and modifiable nucleic acids with no immunogenicity have been FDA-approved for intravenous drugs (*33*, *46*), and the molecular weight of R7-02, the strongest RBD-binding hybrid ligand with the improved nuclease resistance, is just 20 kDa, which is roughly 18% of native hACE2 receptors. Moreover, in choosing the hotspot peptide from hACE2, we intentionally included positively charged and hydrophobic amino acids, i.e., lysine and arginine for the positive charges and leucine and phenylalanine for hydrophobic interactions, to expand the chemical and structural diversity of ligand candidates, which are mainly constituted by hydrophilic and negatively charged nucleic acids. To maximize the synergy of the two biopolymers, peptides and nucleic acids, the evolutionary HOLD process was important for de novo selection of hACE2-mimicking hybrid ligands. As the hotspot interaction with the RBD of SARS-CoV-2 was conserved and even promoted by our hotspot peptide-supporting aptameric scaffold, R7-02 strongly bound to all kinds of SARS-CoV-2 VOCs with binding tolerance, even boosting the affinity by ~500% to Omicron, currently the most mutated, transmissible, and dominant VOC.

Including receptor-like peptides, many kinds of functional peptides and their derivatives have been developed to date, and their synergistic fusion with aptameric scaffolds would be significantly advantageous for many different fields (e.g., drug discovery and delivery, biosensing, and even synthetic biology). For example, despite the presence of various potent peptide drugs, such as insulin, exenatide, and cyclosporine, the relatively low affinity and selectivity to targets have always been a critical issue, which could be well addressed by affinity and selectivity boosting of synergistically combined aptameric scaffolds. Compared to other strategies enabling structural stabilization of peptide drugs (e.g., cyclization by adopting disulfide or thioether bonds, introduction of noncanonical amino acids, and sequence alteration) (*47*), our HOLD technique would provide the most tailored scaffold of certain peptides, not by the complicated rational design, but by the systematic selection process from more than 10^14^ candidates. The aptameric scaffold-driven cooperative binding would be an unrivaled way to maximize the affinity and selectivity of the resulting conjugate, markedly improving the potency and pharmacokinetics of the paired peptide drug. Similarly, the creation of biomarker-targeting peptide-aptamer hybrid ligands would also be highly valuable for targeted drug delivery and early diagnostics, allowing reproducible and scalable chemical synthesis and modification. Moreover, as different modalities can be readily included in the nucleic acid–based scaffold, the peptide-aptamer hybrid ligands could be programmed to perform elaborate actions, such as structure switching (*48*, *49*), catalytic reactions (*50*), target colocalization (*51*), for biosensing, cell signaling, and even synthetic biology applications.

As exemplified by the hACE2-mimicking hybrid ligands, many different receptor mimics could be readily created through our iterative HOLD process when we systematically combine the rational choice of hotspot peptide motif with the in vitro selection of aptameric scaffolds. Recently, protein crystallography is rapidly progressing because of the advance of high-throughput crystallization screening and spectroscopic observation by cryo-EM, thereby allowing newly emerging viruses to be structurally analyzed for hotspot identification just within a month (*52*, *53*). Moreover, by simply correlating the genotype with the phenotype, nucleic acid–based evolutionary techniques are continuously accelerated to discover a variety of functional oligonucleotides, including aptamers (*54*) and nucleozymes (*55*), in high throughput; by automatic screening (*56*), machine learning–guided selection (*57*), and multiparallel characterization (*58*), the in vitro evolution of nucleic acids has been substantially boosted to be completed even in a week (*57*). Furthermore, the peptides and the nucleic acids are chemically synthesizable, ensuring the mass production of peptide-oligonucleotide conjugates with a minimum batch-to-batch variation. Given the technical advances, our HOLD technique is envisioned to easily and quickly create receptor-mimicking hybrid ligands, especially for next-generation viral neutralizers, capable of rapidly coping with potential pandemic situations, regardless of continuously occurring unknown and lethal mutations of infectious viruses.

## MATERIALS AND METHODS

### Materials

DNA oligonucleotides were synthesized, modified, and purified by Bioneer (Korea). The sequence of all the oligonucleotides used in this study is listed in table S2. C-terminal (4-azidobutanoyl(lysine))-tagged LGKGDFR peptides [with and without N-terminal fluorescein isothiocyanate (FITC) modification] were synthesized and purified by Peptron (Korea). 1-Ethyl-3-(3-dimethylaminopropyl) carbodiimide hydrochloride (EDC), *N*-hydroxysuccinimide (NHS), 1 M magnesium chloride solution, hACE2, bovine serum albumin (BSA), high-glucose Dulbecco’s modified Eagle’s medium (DMEM), Tween 20, dimethyl sulfoxide (DMSO), tris(3-hydroxypropyl-triazolylmethyl)amine (THPTA), and copper(II) sulfate pentahydrate were obtained from Sigma-Aldrich. l-Ascorbic acid sodium salt was obtained from Alfa Aesar. Lambda exonuclease enzyme and 10× lambda exonuclease buffer were obtained from Thermo Fisher Scientific. Tris-HCl (1 M, pH 7.4) was obtained from Bioneer (Korea). Phosphate-buffered saline (PBS) (10×), 8 M urea solution, 10× Tris-borate-EDTA (TBE), and nuclease-free water were obtained from T&I (Korea). PBSMT (1×) was used as a binding buffer [consisted of 1× PBS (pH 7.4) containing 2.5 mM MgCl_2_ and 0.02% Tween 20 (v/v)]. SARS-CoV-2 RBDs of wild type, Alpha (B.1.1.7), Beta (B.1.351), Gamma (P.1), and Delta (B.1.617.2) were expressed and purified as described in Supplementary Text. SARS-CoV-2 RBD of Omicron (B.1.1.529) was obtained from Abbexa (United Kingdom). Alexa Fluor 488–labeled monoclonal RBD-binding antibody (P05DHu) and Alexa Fluor 488 microscale protein labeling kit were obtained from Invitrogen. Monoclonal RBD-binding antibody (AM122) was obtained from ACROBiosystems. Macrocyclic peptide (Peptide 4) with N-terminal chloroacetyl-modified d-tyrosine was linearly synthesized by Peptron (Korea). One milligram of the linear Peptide 4 was cyclized by dissolving in 86 μl of DMSO with 3 μl of *N*,*N*-diisopropylethylamine and incubated in 60°C for 24 hours.

### Site-specific conjugation between azide-tagged hotspot peptide and hexynyl-modified ssDNA

For highly efficient copper-catalyzed alkyne-azide cycloaddition, 1 μl of 50 mM copper(II) sulfate in water and 1.67 μl of 150 mM THPTA in water were premixed 10 min before the conjugation. Three microliters of DMSO, 1 μl of 10× PBS, 3 μl of 0.1 mM hexynyl-modified ssDNA in water, 1 μl of 2.5 mM azide-tagged LGKGDFR peptide, 2.67 μl of premix [copper(II) sulfate and THPTA], and 1 μl of 500 mM sodium ascorbate solution in water (to be freshly prepared) were added in order and completely mixed. The solution was shaken at room temperature overnight and purified by ethanol precipitation.

### Confirmation of the peptide-ssDNA conjugated hybrid library

The formation of peptide-ssDNA conjugates was fluorescently verified by coupling FITC-modified azide-tagged LGKGDFR peptide. One microliter of the reacted products (~50 ng), 7 μl of 8 M urea solution, and 1 μl of 6× loading dye were mixed and heat-denatured at 95°C for 10 min. The denatured products were analyzed via electrophoresis on a 10% urea polyacrylamide gel in 1× TBE buffer at 300 V for 40 min and imaged by Azure C600 (Azure Biosystems). The same amount of unconjugated hexynyl-modified ssDNA library was stained with 10,000× SYBR gold staining dye (Invitrogen) and imaged in parallel (fig. S1).

### HOLD process

A hybrid random library was heat-denatured at 95°C for 10 min and then snap-cooled in ice to ensure the formation of the most stable 3D structures. This heat-treated hybrid library was then incubated for 1 hour at room temperature with the RBD-coated beads (table S1 for selection parameters) with gentle rotation. In preparing the RBD-coated beads, target RBD was immobilized onto the Dynabeads M-270 carboxylic acids (Invitrogen) through the EDC-NHS coupling process using the manufacturer’s protocol, followed by quantification with the NanoOrange Protein Quantitation Kit (Thermo Fisher Scientific). After the incubation, the magnetic beads were thoroughly washed with 1× PBSMT to eliminate unbound hybrid molecules (table S1 for washing conditions), and magnetic bead-bound hybrid ligands were selectively isolated using a DynaMag-2 magnet (Invitrogen). Only the DNA domain of the isolated hybrid ligand candidates was PCR-amplified with the 5′-hexynyl–modified forward primer and 5′-phosphorylated reverse primer (table S2). At every round of PCR, the optimal PCR cycle number was decided by the pilot PCR process. The PCR-amplified double-stranded DNAs (dsDNAs) were purified using the QIAquick PCR purification kit (QIAGEN) and then digested with lambda exonuclease to generate hexynyl-modified ssDNA pools for the next round of HOLD. The ssDNAs were purified by phenol/chloroform extraction and ethanol precipitation and quantified by ultraviolet-visible (UV-vis) measurement at 260 nm. To provide the same format of hybrid library pool for every round of HOLD, the click reaction between the azide-tagged LGKGDFR peptide and the purified hexynyl-modified ssDNA pool was repeated for every round of HOLD.

### Volume dilution challenge for a highly stringent condition of HOLD

The volume dilution challenge technique is used for the highly efficient removal of unbound hybrid molecules (*59*). After 1 hour of incubation and subsequent washing of bead-bound hybrid library, they were immediately diluted into the increased volume (100 to 500×) of 1× PBSMT and incubated for an additional 30 min to remove hybrid molecules that were easily detached in this process due to the high dissociation constant (*k*_off_) value. After the volume dilution challenge, the beads were collected and washed three times with 200 μl of 1× PBSMT.

### DNA domain amplification to enrich high-affinity hybrid ligand

The 100-μl PCR reaction mixture contained 5 U of Taq polymerase (T&I, Korea), 0.5 μl of 0.1 mM 5′-hexynyl–modified forward primers and 5′-phosphorylated reverse primers, 8 μl of 10 mM deoxynucleoside triphosphate (T&I, Korea), 2 μl of 10× PCR buffer (T&I, Korea), 10 μl of collected hybrid ligands, and additional nuclease-free water (up to 100 μl). The PCR reaction mixture was predenatured at 95°C for 600 s, followed by repeated cycles of 30 s of denaturation at 95°C, 30 s of annealing at 51°C, and 60 s of extension at 72°C. Five microliters of PCR mixture was collected and resolved on 10% polyacrylamide gel to determine the optimal PCR amplification cycle number with minimal by-products (pilot PCR). DNA domains of the collected hybrid ligands from every round of HOLD were PCR-amplified at the optimized cycle number.

### ssDNA generation

After the PCR amplification, hexynyl-modified dsDNAs were purified using the QIAquick PCR Purification Kit (QIAGEN), and 2.5 μg of the purified dsDNA was digested with 5 U of lambda exonuclease (Thermo Fisher Scientific) in 50 μl. After incubating the mixture for 10 min at 37°C and 10 min at 80°C, the hexynyl-modified ssDNAs were purified by phenol/chloroform extraction and ethanol precipitation and quantified by UV-vis measurement at 260 nm.

### Relative binding assay for R7 pool

After seven rounds of HOLD, we compared the relative binding capability of the populated hybrid ligand pool. The same amount of random DNA library coupled with the hotspot peptide, R7 ssDNA pool without the hotspot peptide, and R7 ssDNA pool coupled with the hotspot peptide were heat-denatured at 95°C for 10 min and then snap-cooled in ice. After heat denaturation, they were challenged with RBD-coated magnetic beads for 1 hour at room temperature and subsequently washed three times with 1× PBSMT. The amount of RBD binding was analyzed by quantitative PCR (LightCycler 480, Roche). Each PCR reaction contained 10 μl of LightCycler 480 SYBR green I master (Roche), 0.1 μl of 0.1 mM forward primers and reverse primers, 2 μl of RBD-bound ligands, and 7 μl of nuclease-free water. On the basis of the standard curve we have previously obtained, the threshold cycles were extrapolated to the quantities of RBD-bound ligands (fig. S6).

### High-throughput sequencing

After seven rounds of selection, the enriched pool was PCR-amplified with forward and reverse primers that includes adaptor sequences (table S2), and an optimized PCR cycle number was determined by pilot PCR. These PCR products were purified using a gel extraction kit (QIAGEN) and subsequently sequenced with Illumina MiSeq Next-Generation Sequencing at the SYSGENLAB (Korea). Sequencing library was analyzed in 101–base pair PE (paired-end) sequencing mode on the Illumina NovaSeq 6000 platform. The sequencing reads quality was measured by FastQC tool, and the adapter sequences were removed by cutadapt trimmer with the -e 0.1 -j 20 option. Only perfectly matched-paired reads were used for further analysis. The high-quality sequencing reads passed the fastq_quality_filter (≥Q30) from the FASTX toolkit (RRID:SCR_005534).

### Affinity and specificity analysis of the hybrid ligand

To assess the binding affinity of the hybrid ligand toward the wild-type RBD and its variants, the DNA sequence of R7-02 was synthesized with the 5′-hexynyl group for hotspot peptide conjugation and 3′-FAM for flow cytometry–based binding analysis. The FAM-labeled R7-02 in 1× PBSMT was heat-denatured and snap-cooled as explained above. After heat denaturation, varying concentrations of R7-02 were challenged with RBD-coated beads for 1 hour at room temperature with gentle rotation. The beads were washed twice with 1× PBSMT to rule out nonspecific binding, and bead-bound R7-02 was then collected using a DynaMag-2 magnet (Invitrogen). The collected beads were resuspended in 1× PBSMT and analyzed by measuring the mean fluorescence of beads using CytoFLEX S (Beckman Coulter). For each measurement, 15,000 events were analyzed. Last, *K*_d_ was calculated using nonlinear regression analysis (Prism software, GraphPad Prism version 9.3.1). To assure their binding specificity, a similar characterization process was conducted for the beads coated with BSA and noncoated beads (bare beads) in parallel. In preparing the noncoated beads, Dynabeads M-270 carboxylic acid beads were tris-blocked for 15 min in 50 mM tris-HCl (pH 7.4) for 15 min at room temperature. Similar characterization processes were conducted for highly populated sequence families (R7-01 and R7-03) and the reported RBD-binding affinity reagents: Aptamer-1, CoV2-RBD-1C, CoV2-6C3, Peptide 4, P05DHu, and AM122 (figs. S7 and S8).

### Molecular docking simulation of R7-02 in complex with RBD

To rationalize the high binding affinity of R7-02 to RBD, the molecular structure of R7-02 in complex with the RBD was constructed using molecular docking simulation. The crystal structure of the hACE2-derived hotspot peptide, LGKGDFR, and the single RBD of SARS-CoV-2 spike protein were obtained from the Research Collaboratory for Structural Bioinformatics (RCSB) Protein Data Bank (PDB) (PDB ID: 6M0J), with a single site mutation (N501Y) of RBD. The secondary structure of the aptameric scaffold in R7-02 (table S2) was predicted using the mfold web server, which was fed into the RNAComposer for 3D construction. The peptide-aptamer hybrid structure of R7-02 was eventually constructed by attaching a triazole-modified linker residue between the hexynyl-modified 5′ end of the folded DNA and the butanamide-modified lysine C terminus of the hotspot peptide. Molecular docking simulation was subsequently performed to find the best fit of the hotspot peptide-bound aptameric scaffold to the RBD, while constraining the hotspot peptide and the RBD in their crystal position. The resulting structure was energy-minimized using the AMBER14 force field, using a molecular dynamics simulation package OpenMM version 7.4.

### Competitive binding assay with reported RBD-binding affinity reagents

For the competitive binding assay, R7-02 and the reported RBD-binding aptamers were modified with FAM and tetramethylrhodamine (TAMRA), respectively (table S2). After heat denaturation and snap-cooling as explained above, they were co-incubated with 5 nM RBD beads (final volume: 100 μl) for 1 hour at room temperature with gentle rotation. The beads were washed three times and resuspended in 100 μl of 1× PBSMT to be analyzed by CytoFLEX S (Beckman Coulter) after signal compensation. The competitive RBD-binding fraction was calculated by the ratio of the mean fluorescence intensity with competition to the mean fluorescence intensity without competition (free binding). For the competitive binding with the reported RBD-binding cyclic peptide and antibodies, TAMRA-modified R7-02 is co-incubated with FAM-modified peptide or Alexa Fluor 488–modified RBD antibodies and analyzed in the same method.

### Binding fraction comparison between the wild-type and the Omicron RBD

To analyze the binding tolerance to the SARS-CoV-2 variants among the chosen RBD binders (Aptamer-1, Peptide 4, P05DHu, and AM122), we quantitatively measured the binding fractions for the wild-type and the Omicron RBD at 10 nM R7-02 and the antibodies (P05DHu and AM122) and at 100 nM aptamer (Aptamer-1) and the cyclic peptide (Peptide 4). After 1 hour of incubation of RBD-coated magnetic beads with each binder at room temperature with gentle rotation, the beads were washed three times and resuspended in 100 μl of 1× PBSMT. The binding fractions of each binder to the wild-type and the Omicron RBD were directly measured by CytoFLEX S (Beckman Coulter).

### ELISA-based RBD-hACE2 binding inhibition test

In characterizing the inhibition efficiency, a SARS-CoV-2 inhibitor screening kit (ACROBiosystems) was used according to the manufacturer’s protocol. The microplate has been precoated with hACE2, and the varying concentrations of R7-02 (1 pM to 1 μM) were added to the plate followed by the immediate addition of horseradish peroxidase–conjugated SARS-CoV-2 wild-type RBD. After 1 hour of incubation at room temperature, the wells were washed three times, and substrates were added to quantify the RBD-hACE2 binding. The reaction was terminated by the addition of the stop solution, and the intensity of absorbance at 450 nm was measured using a microplate reader (Tecan). The IC_50_ value for the R7-02–driven inhibition of the RBD-hACE2 binding was determined after log transformation of hybrid ligand concentrations using sigmoidal dose-response nonlinear regression analysis (Prism software, GraphPad Prism version 9.3.1). In the same way, the RBD-hACE2 inhibition was compared after adding 3 μM individual ligands (LGKGDFR peptide, DNA domain of R7-02, and R7-02) to analyze the synergistic interplay between the hotspot peptide and the aptameric scaffold in inhibiting the RBD-hACE2 interaction.

### Cell culture and sample preparation

The hACE2-293T cell line was obtained from Takara (Japan). hACE2-293T cells were cultured at 37°C, 5% CO_2_ atmosphere, with high-glucose DMEM (Sigma-Aldrich) supplemented with 10% fetal bovine serum (Gibco) and 1% sodium pyruvate (Sigma-Aldrich). Five to 15 passages of hACE2-293T were used for the pseudotyped SARS-CoV-2 neutralization assay.

### Pseudotyped SARS-CoV-2 neutralization assay

To determine the neutralization ability of R7-02, the pseudotyped SARS-CoV-2 neutralization assay was performed. The hACE2-293T cells (~4 × 10^3^) were preseeded into 96-well plates and cultured overnight. After 24 hours, 5 μl of GFP-reporting pseudotyped SARS-CoV-2 (4 × 10^5^ transducing unit/ml; BPS Bioscience) was preincubated with 3 μM R7-02 in 1× PBSM (1× PBS with 2.5 mM magnesium) for 30 min at room temperature. The cell culture medium in preseeded hACE2-293T cells was removed, and the cells were washed twice with prewarmed 1× PBSM. After washing, the mixture of pseudovirus and R7-02 was added to the cells and infected at 37°C for 6 hours. After the infection, the 1× PBSM was replaced with fresh cell culture medium, and the cells were further cultured at 37°C for another 48 hours. Last, GFP fluorescence images and the bright-field images were observed using confocal microscopy (Leica DMi8; source wavelength: 488 nm). The percentage of GFP-expressing infected cells over the total hACE2-293T cells was computationally analyzed by CellProfiler version 4.2.1 (Fig. 4E and fig. S11). In the same way, the neutralizing efficacy of other RBD-binding ligands (Aptamer-1, CoV2-RBD-1C, CoV2-6C3, P05DHu, AM122, and Peptide 4) was analyzed.

### Serum stability test

To characterize serum stability, we allowed nucleases to degrade the FAM-labeled R7-02 for up to 24 hours in 10% fetal bovine serum at 37°C. For comparison, R7-02 with other modifications, such as the introduction of phosphorothioate backbones and 2′-*O*-methyl and 2′-F modification (see details in table S2), and other unmodified ssDNAs were tested together. Four microliters of 10 μM samples was mixed with 10× PBS (4 μl), fetal bovine serum (4 μl), and nuclease-free water (28 μl). After 0, 0.5, 2, 4, 8, 18, and 24 hours of incubation at 37°C, 5 μl of each aliquot from the different mixtures was immediately treated in 90°C for 5 min and stored in ice before analysis. The aliquots were analyzed via electrophoresis on a 10% urea polyacrylamide gel in 1× TBE buffer and imaged by Azure C600 (Azure Biosystems) after staining with 10,000× SYBR gold staining dye (Invitrogen).

## Supplementary Materials

### This PDF file includes:

Supplementary Text Figs. S1 to S13 Tables S1 to S4 References

## Notes

View/request a protocol for this paper from *Bio-protocol*.
