# Supplementary material for: De novo selected hACE2 mimics that integrate hotspot peptides with aptameric scaffolds for binding tolerance of SARS-CoV-2 variants
Source: Sci Adv. 2022 Oct 26;8(43):eabq6207. doi: 10.1126/sciadv.abq6207 (PMC9604513; doi:10.1126/sciadv.abq6207)
Supplement: Supplementary file 1 — Supplementary Text Figs. S1 to S13 Tables S1 to S4 References [file sciadv.abq6207_sm.pdf]

Science Advances
AAAS

Supplementary Materials for

De novo selected hACE2 mimics that integrate hotspot peptides with aptameric scaffolds for binding tolerance of SARS-CoV-2 variants

Minjong Lee *et al.*

Corresponding author: Seung Soo Oh, seungsoo@postech.ac.kr

Sci. Adv. **8**, eabq6207 (2022)
DOI: 10.1126/sciadv.abq6207

This PDF file includes:

Supplementary Text
Figs. S1 to S13
Tables S1 to S4
References

Supplementary Text

Construction of plasmids to express wild-type RBD and RBD variants in mammalian cells. All plasmids and primers used in this study are described (table S3 and S4). The wild-type SARS-CoV-2 RBD gene synthesized by assembly of a list of primers (JS#162-175) was PCR amplified using two primers (JS#162/JS#176) and ligated into pMAZ-IgH-GlycoT using XbaI and BssHII restriction endonuclease sites to generate pMAZ-SARS-CoV-2-WT-RBD-His. Mutations for RBD variants, Alpha (B.1.1.7), Beta (B.1.351), Gamma (P.1), and Delta (B.1.617.2), were introduced into pMAZ-SARS-CoV-2-WT-RBD-His using QuikChange II site-directed mutagenesis kit (Agilent Technologies) and each set of primers (JS#179/JS#180 for N501Y mutation, JS#181/#182 for K417N mutation, JS#183/#184 for E484K mutation, JS#185/#186 for K417T, JS#187/#188 for L452R, and JS#191/JS#192 for T478K mutation) to generate pMAZ-SARS-CoV-2-Alpha (B.1.1.7)-RBD-His, pMAZ-SARS-CoV-2-Beta (B.1.351)-RBD-His, pMAZ-SARS-CoV-2-Gamma (P.1)-RBD-His, and pMAZ-SARS-CoV-2-Delta (B.1.617.2)-RBD-His, respectively.

Preparation of wild-type RBD and RBD variants in mammalian cells.
For transient expression of RBD variant, 300 μg of purified plasmids (pMAZ-SARS-CoV-2-WT-RBD-His, pMAZ-SARS-CoV-2-Alpha (B.1.1.7)-RBD-His, pMAZ-SARS-CoV-2-Beta (B.1.351)-RBD-His, pMAZ-SARS-CoV-2-Gamma (P.1)-RBD-His, or pMAZ-SARS-CoV-2-Delta (B.1.617.2)-RBD-His) were transfected into 300 mL of Expi293F cells (2×10^6 cells/mL) using 1.2 mg of polyethylenimine (Polysciences, Taipei, Taiwan). After growing transfected cells in FreeStyleTM expression medium (Thermo Fisher Scientific) for 6 days and pelleting cells by centrifugation at 4,000x g for 10 min, the media was mixed with 12.5 mL of 25x PBS and filtered through a 0.2 μm bottle top filter (Thermo Fisher Scientific). Then, the filtrate was incubated with 1 mL of Ni-NTA agarose resin (Qiagen) at 4°C for overnight and passed through a polypropylene column (Thermo Fisher Scientific). After washing Ni-NTA agarose resin in 50 mL of 1x PBS, 25 mL of wash buffer A (10 mM imidazole in 1x PBS), and 25 mL of wash buffer B (20 mM imidazole in 1x PBS) sequentially, RBD proteins were eluted using 5 column volumes of elution buffer (200 mM imidazole in 1x PBS). After exchanging the buffer with 1x PBS and concentrating using Amicon Ultra-4 spin column (Merck Millipore), the purities of RBD proteins were assessed by running on a 4-20% SDS-PAGE gel (fig. S13A).

To verify activities of purified RBD proteins, 50 μL of 4 μg/mL of RBD (wild-type, Alpha, Beta, Gamma, and Delta) diluted in 0.05 M Na_2CO_3 (pH 9.6) were coated onto a flat-bottom polystyrene high-bind 96 well microplate (Costar, Washington D.C., USA) by incubating at 4°C for overnight. After blocking with 4% skim milk, 1x PBS for 2 hours at room temperature and washing four times in 180 μL of PBST (1x PBS, 0.05% Tween20, pH 7.4), the plate was added with 50 μL of CR30221, an anti-SARS-CoV-2-RBD IgG antibody, which was prepared in house and serially diluted in the 1% skim milk. After incubation at room temperature for an hour and four times washing in PBST, 50 μL of 1:5,000 diluted goat anti-human IgG (H+L) antibody-HRP conjugate (Thermo Fisher Scientific) was added. After four times washing in PBST, addition of 50 μL of 1-Step Ultra TMB-ELISA Substrate Solution (Thermo Fisher Scientific), and quenching the colorimetric reaction with 50 μL of 4 N of H_2SO_4, the ELISA binding signals were detected by measuring the absorbance at 450 nm using an Epoch microplate spectrophotometer (BioTek, Winooski, VT, USA) (fig. S13B).

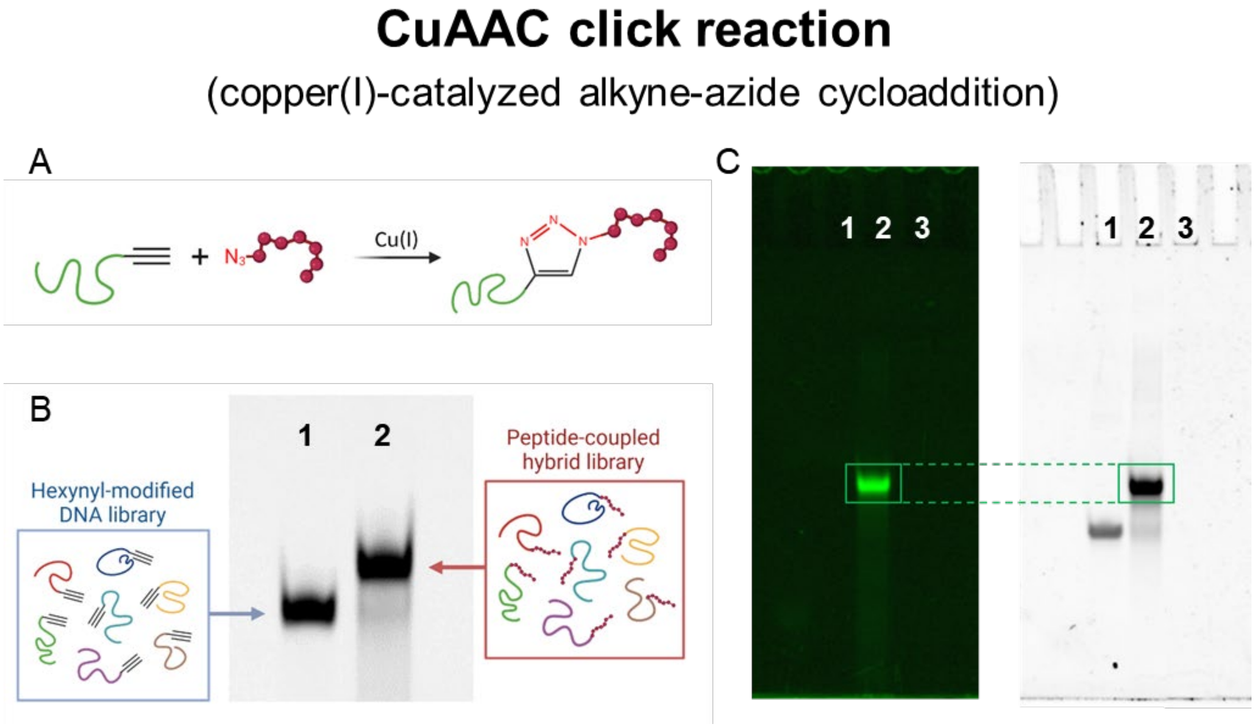

Fig. S1.

Confirmation of peptide-ssDNA conjugation for a hybrid random library. (**A**) Schematic representation of peptide-ssDNA conjugation through CuAAC. 5'-hexynyl-modified ssDNA (green) and C-terminus-azide-tagged hot-spot peptide (red) can be site-specifically conjugated to form unique hybrid architecture. (**B**) Denaturing polyacrylamide gel electrophoresis (PAGE) to confirm the hot-spot peptide conjugation. Lane 1: Hexynyl-modified ssDNA library. Lane 2: Peptide-coupled hybrid library. Only the DNA motifs were stained and imaged. (**C**) Fluorescent confirmation of the hot-spot peptide conjugation. Green channel imaging (left) and ssDNA-stained imaging (right) within identical electrophoresed gel. Lane 1: Simple mixture of hexynyl-modified DNA library and hot-spot peptide. Lane 2: Peptide-coupled hybrid library. Lane 3: FITC peptide only. Even without ssDNA staining, the hybrid library is confirmed by FITC-coupling.

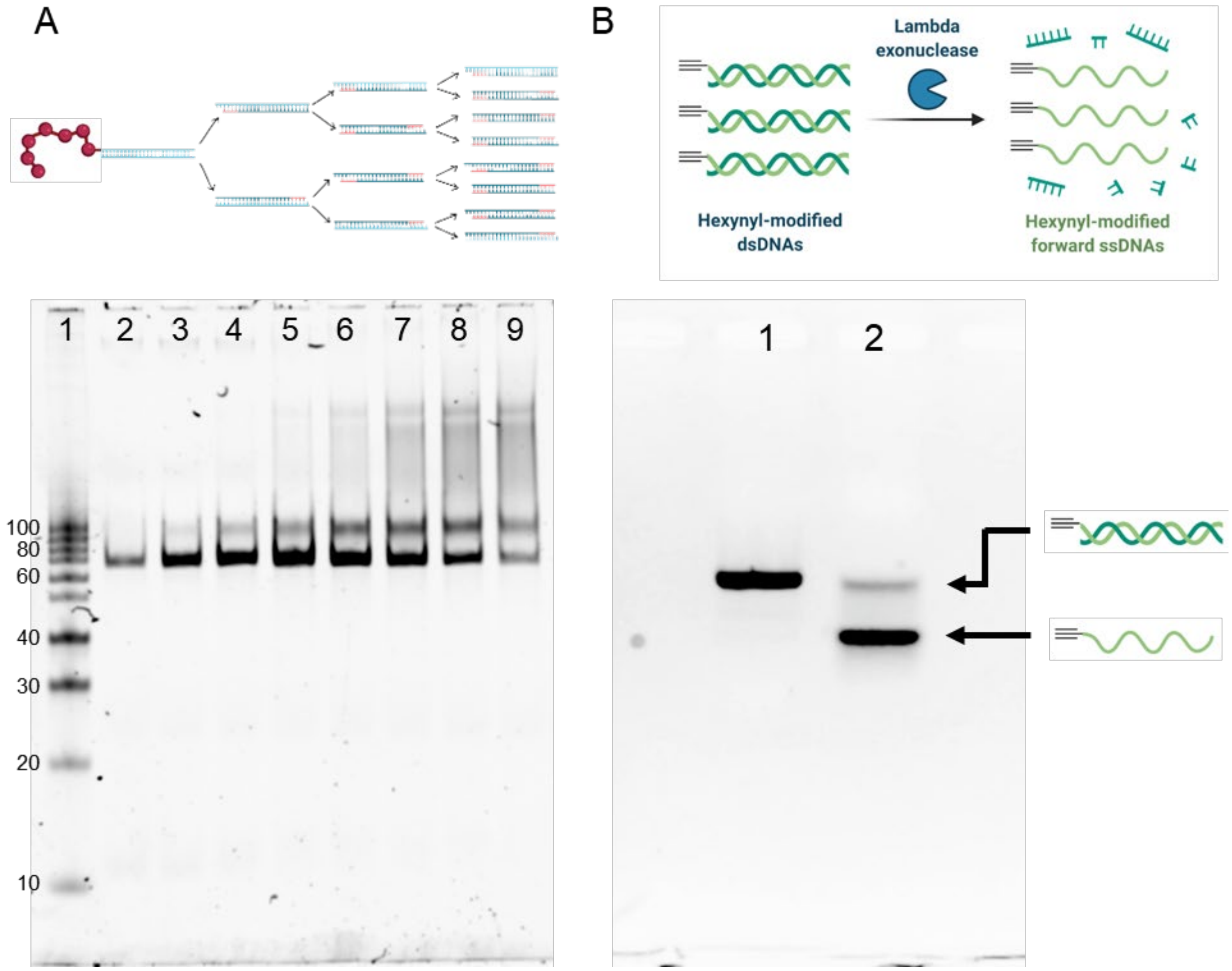

Fig. S2.

Validation of the amplification and purification steps of HOLD. (**A**) Even with the coupled hot-spot peptide, the 70nt-DNA domain of the hybrid ligands were PCR-amplified well. The amplified products were resolved by PAGE. Lane 1: 10bp DNA Ladder. Lane 2-9: PCR products obtained every other cycle. (**B)** From 5'-hexynyl-modified double-stranded DNA (dsDNA), 5'-hexynyl-modified single-stranded DNA (ssDNA) was separated after lambda exonuclease digestion. ssDNA generation was analyzed by agarose gel electrophoresis. Lane 1: Hexynyl-modified dsDNAs as PCR products. Lane 2: Hexynyl-modified ssDNAs after lambda exonuclease digestion.

A

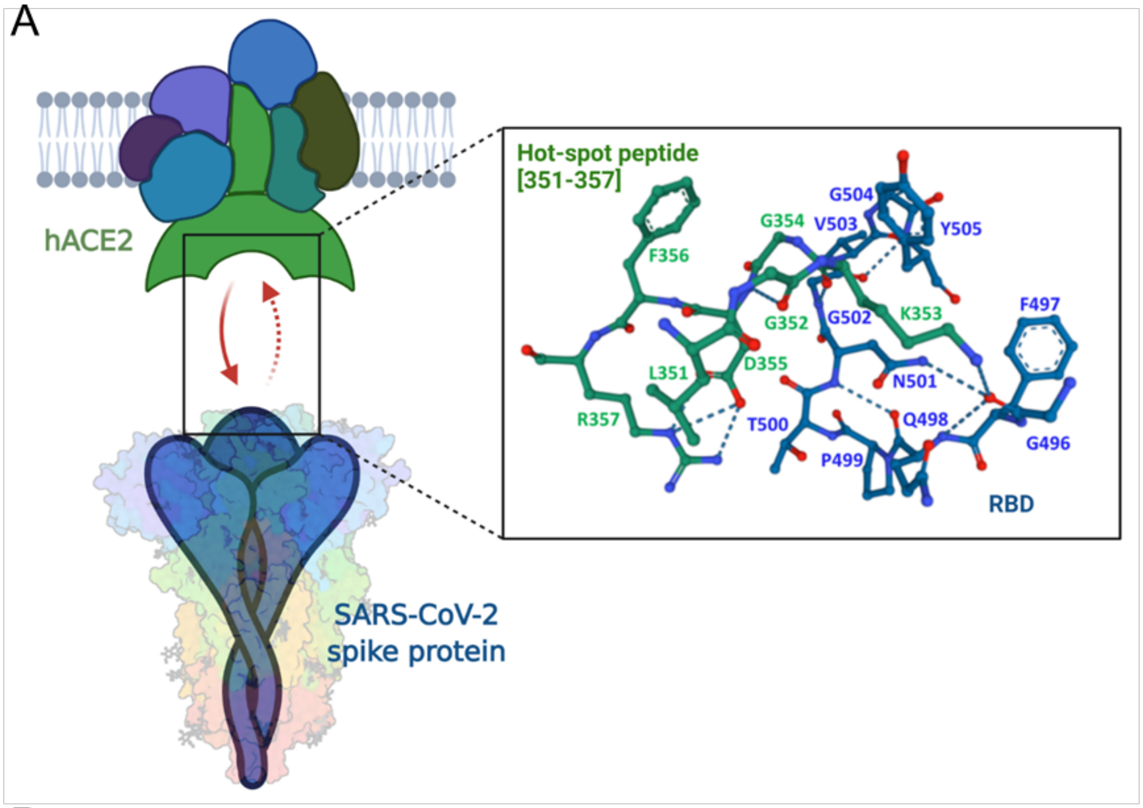

B

hACE2	S19	Q24	T27	F28	K31	H34	E35	E37	D38	Y41	Q42	L45	L79	M82	Y83	Q325	K353	G354	D355	R357
RBD of SARS-CoV-2	A475	G479<br>N487	F459<br>A475<br>Y489	Y489	Y456<br>Q493	Y453<br>L455	Q493	Y505	Y449	Q498<br>T500<br>N501	Q498	Q498	F489	F486	F486<br>N487	T500	G496<br>N501<br>G502<br>Y505	G502<br>Y505	T500	T500

Fig. S3.

Structural analysis of the interface between hACE2 and SARS-CoV-2 RBD. (**A**) Representation for the binding interaction between the hACE2-derived hot-spot peptide and the RBD of SARS-CoV-2. Contact residues of the hACE2 with the RBD were structurally analyzed from PDB 6M0J. In the crystal structure of hot spot interaction region, the hot-spot peptide, L351-R357, and the interaction residues of the RBD are represented as green and blue, respectively. (**B**) List of contact residues from the entire hACE2 that are directly involved in RBD-hACE2 binding (36).

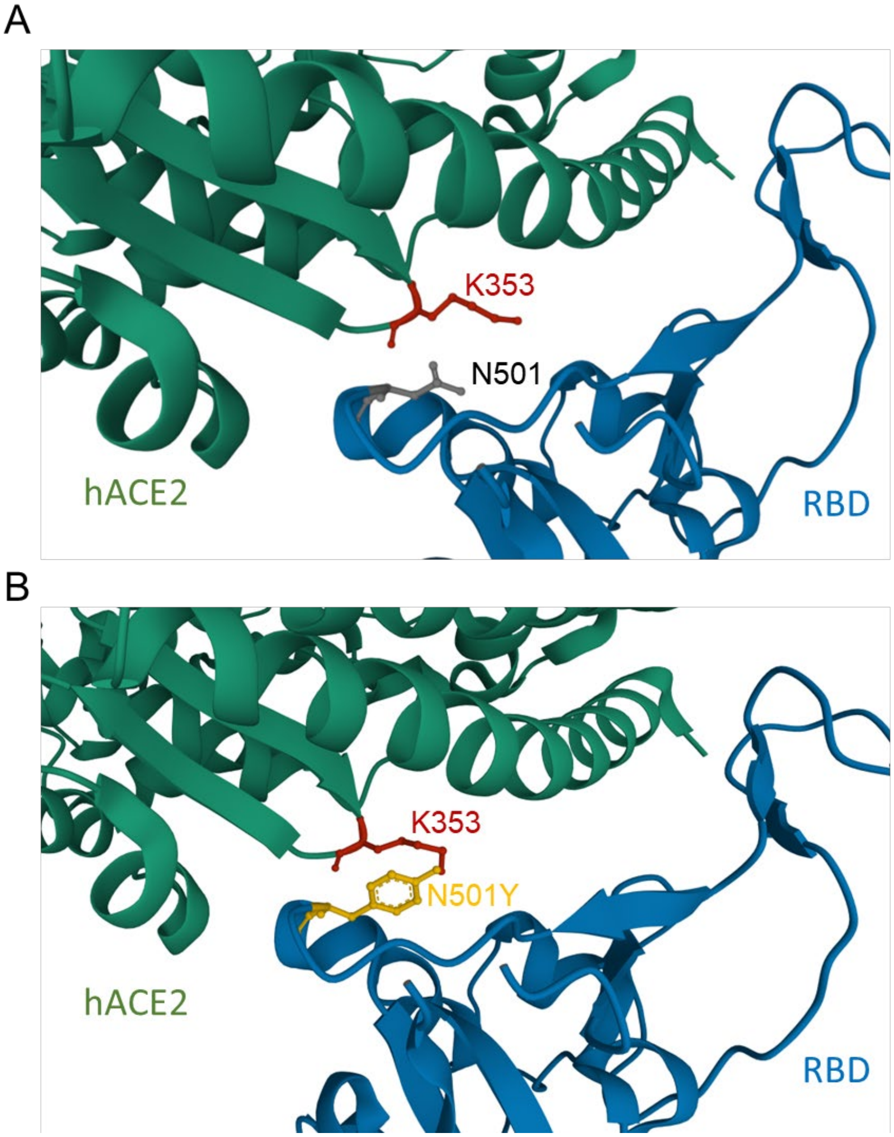

Fig. S4.

N501Y mutation for a higher binding affinity toward hACE2. (**A-B**) The predicted binding of N501 (**A**, gray) and mutated N501Y (**B**, yellow) to the K353 of hACE2 receptor (red). Due to the N501Y mutation of the RBD, the VOCs bind more tightly and strongly to the hACE2 because the K353 of the hACE2 involves in an unexpected hydrophobic interaction with the substituted tyrosine (*7, 8*). Images were structurally analyzed from PDB 7WBP and 6M0J.

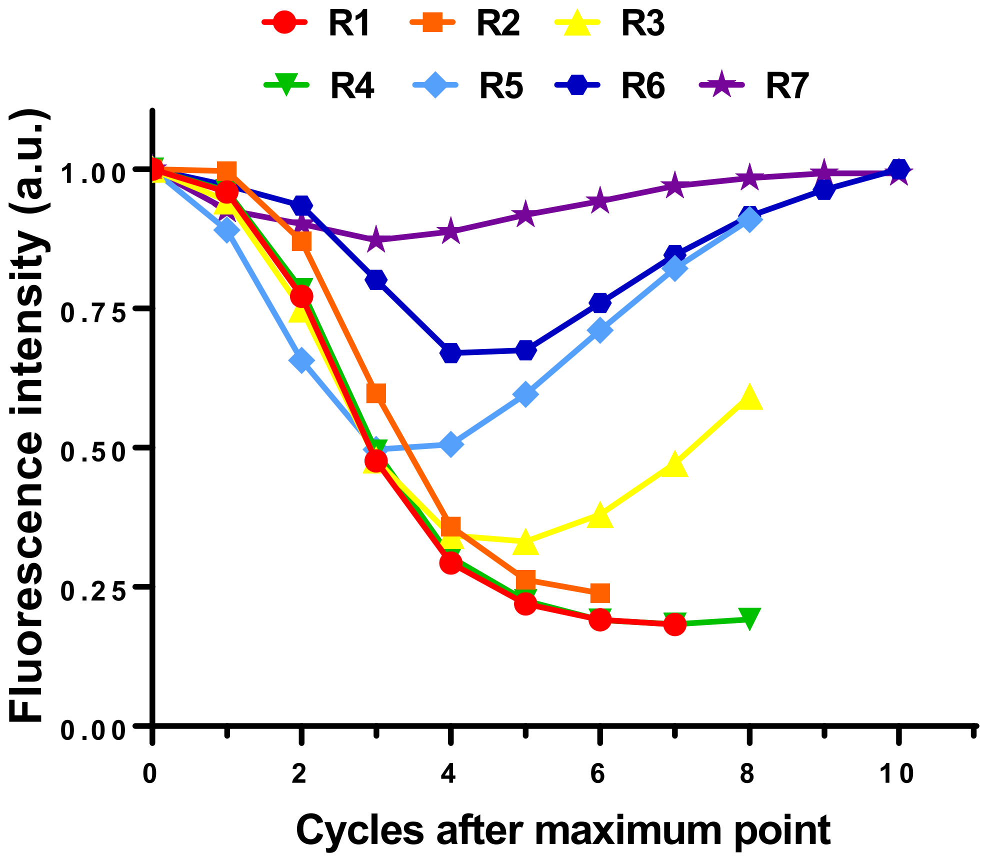

Fig. S5.

Evaluation of the heterogeneous populations of hybrid ligands by the shape of amplification curve. By profiling the qPCR amplification curve for every round of HOLD process, we confirmed that the diversity of hybrid ligand pool has decreased as the HOLD process were repeated. The relative decrease of fluorescence after passing the maximum point is known to be directly proportional to the logarithm of the number of oligonucleotide sequence.

A

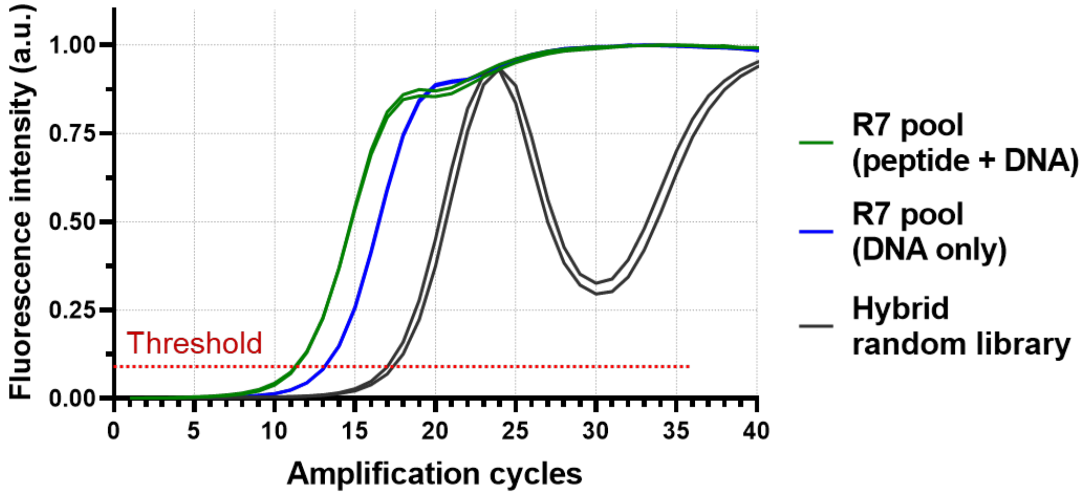

B

Conc. of hexynyl-modified random library	Threshold cycle (C_T)
1 nM	7.91
	8.09
100 pM	11.16
	11.24
10 pM	14.4
	14.6
1 pM	17.85
	17.95

C

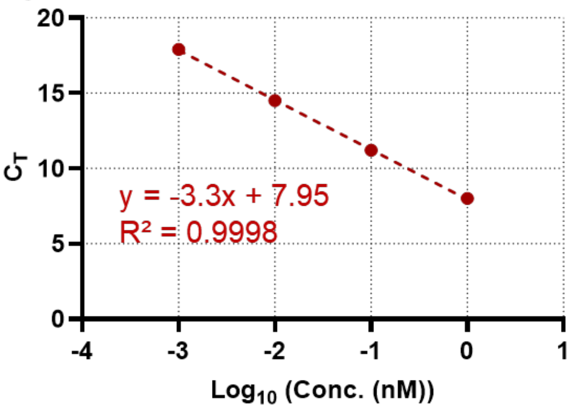

Fig. S6.

qPCR-based relative binding assay to confirm the synergistic interplay of hybrid ligands. (A) After 7 rounds of HOLD, the amplification curves were compared among three different groups: 1) R7 ssDNA pool coupled with the hot-spot peptide (green), 2) R7 ssDNA pool without the hot-spot peptide (blue), and 3) random DNA library coupled with the hot-spot peptide (gray). On the basis of the standard curve, the threshold cycle of each group was extrapolated to the amounts of RBD-bound ligands. (B) For different concentrations of random library (1 nM, 100 pM. 10 pM, and 1 pM), threshold cycles (C_T) were measured by quantitative PCR. (C) By linear regression of hexynyl-modified library concentrations and their C_T values, the amount of RBD-bound ligands in the three different pools could be calculated, and based on the amount of initially introduced RBDs, the corresponding binding fractions were subsequently determined.

A

Copy rank	Sequences (5' → 3')	Number of sequences	Percentage of the sequence family
1	CTCCACAACCGCTGATCGACGCAGATCTTGTGGGT	483,860	14.416 %
	CTCCACAACCGCTGATCGACGCAGATCTTGTGGT	16,249	
2	ACGCAATCGTGGGTTGGGGATATAATGGTA	35,997	1.834 %
	ACGCAATCGTGGGTTGGGGACATAATGGTA	13,909	
	ACGCAATCGTGGGTTGGGATATAATGGTA	13,703	
3	CACACCAAATCTGAGTCTACCCCATGAAGG	30,943	1.769 %
	CACACCAAATCTGAGTCTACCCTATGAAGG	30,433	

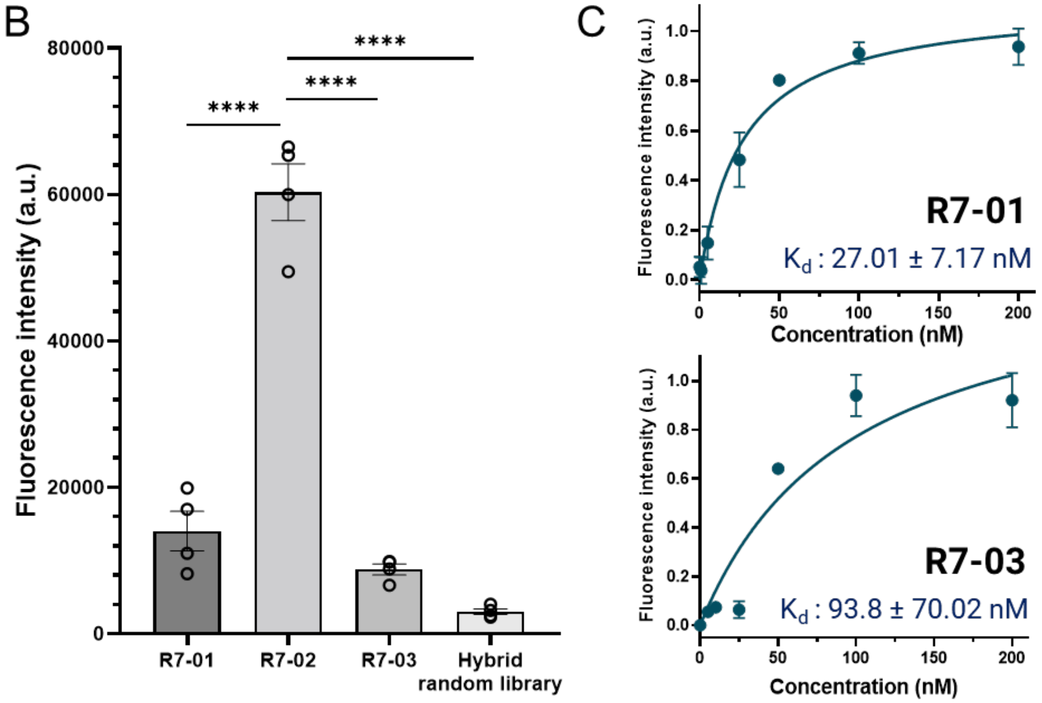

Fig. S7.

HTS results for the sequence of potent peptide-supporting aptameric scaffolds. (**A**) From the R7 pool populated with potent hybrid ligands, the three most abundant sequence families were revealed from high-throughput sequencing (HTS). The sequence of random region is represented. (**B**) Relative binding assay between the highly populated hybrid ligand. RBD binding-dependent fluorescence intensity is compared between top three abundant hybrid ligand (R7-01, R7-02, and R7-03). n=4 biological replicates (****p<0.0001; bars represent means ± s.e.m). (**C**) Affinity characterization of R7-01 and R7-03. The RBD binding-dependent fluorescence intensity was plotted in varying concentrations of R7-01 and R7-02 (0-200 nM) for calculation of equilibrium dissociation constant (K_d). 15,000 beads were analyzed for each measurement (bars represent means ± s.d).

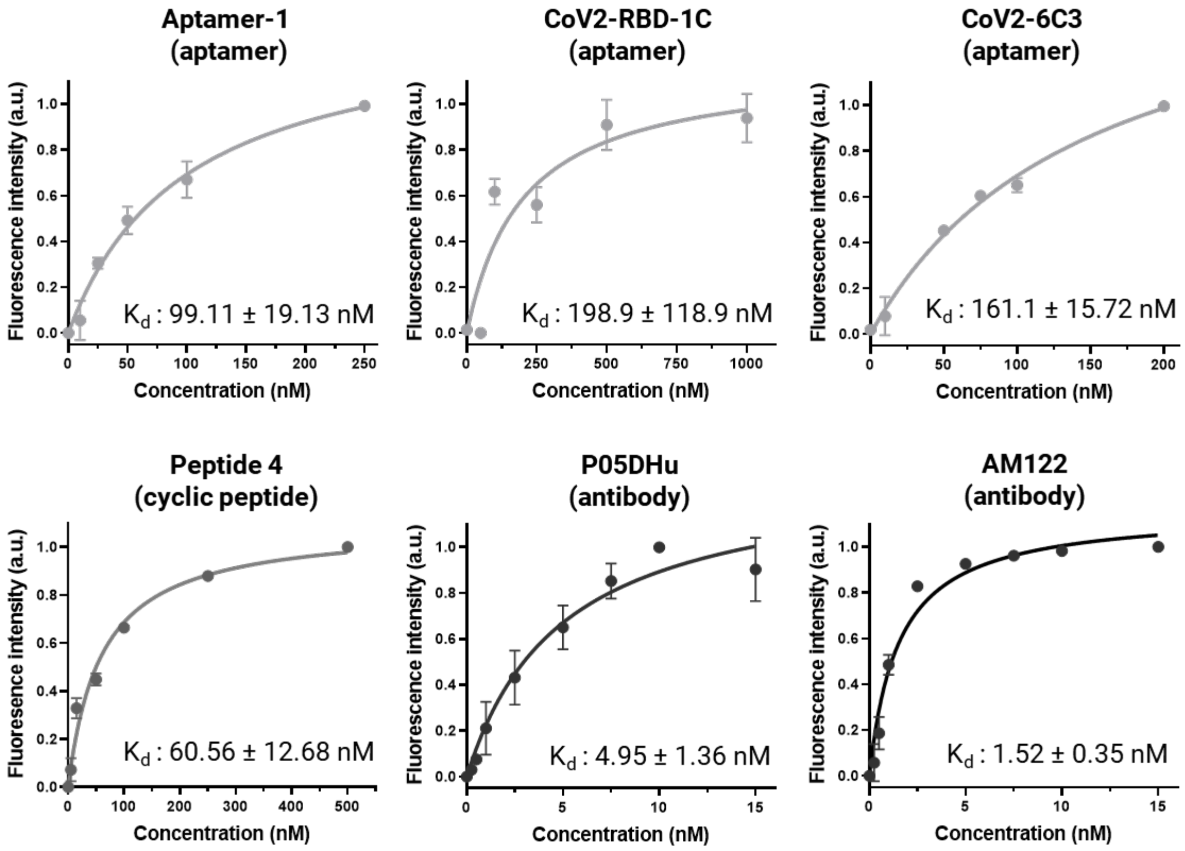

Fig. S8.

Affinity characterization of previously reported RBD binders. The RBD binding-dependent fluorescence intensity was plotted in varying concentrations of previously reported RBD binders for calculation of equilibrium dissociation constant (K_d). 15,000 beads were analyzed for each measurement (bars represent means ± s.d).

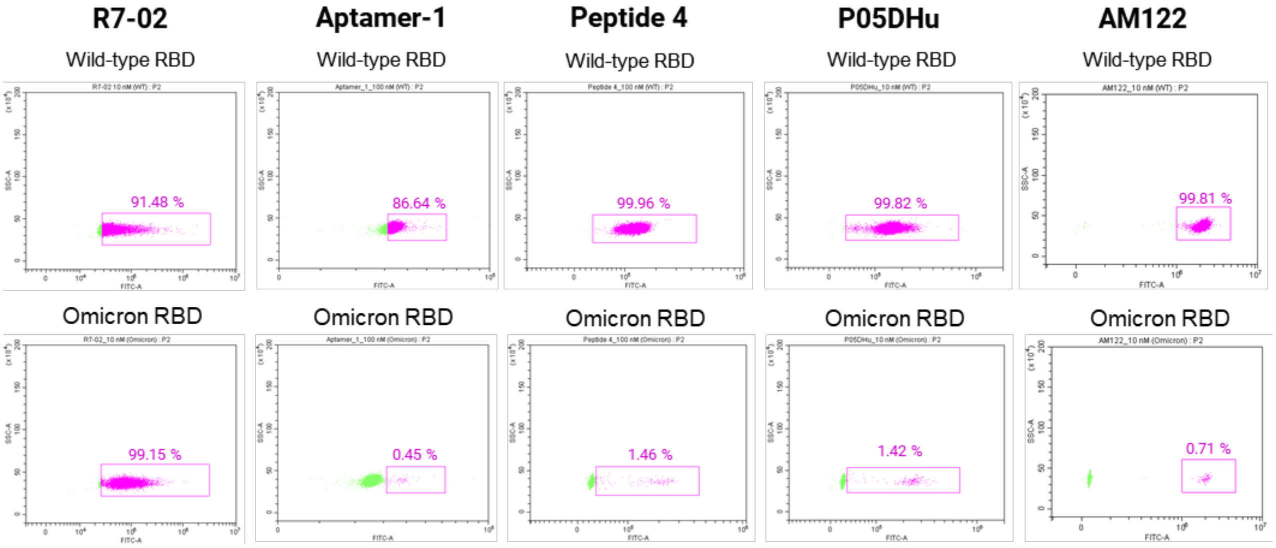

Fig. S9.

Comparison of different RBD affinity reagents in binding the wild type and the Omicron RBDs. Using a flow cytometer (CytoFLEX S, Beckman Coulter) we counted the number of RBD-coated beads in complex with each fluorophore-labeled affinity reagents to directly measure the binding fractions for the wild type and the Omicron RBD. 15,000 beads were analyzed for each measurement.

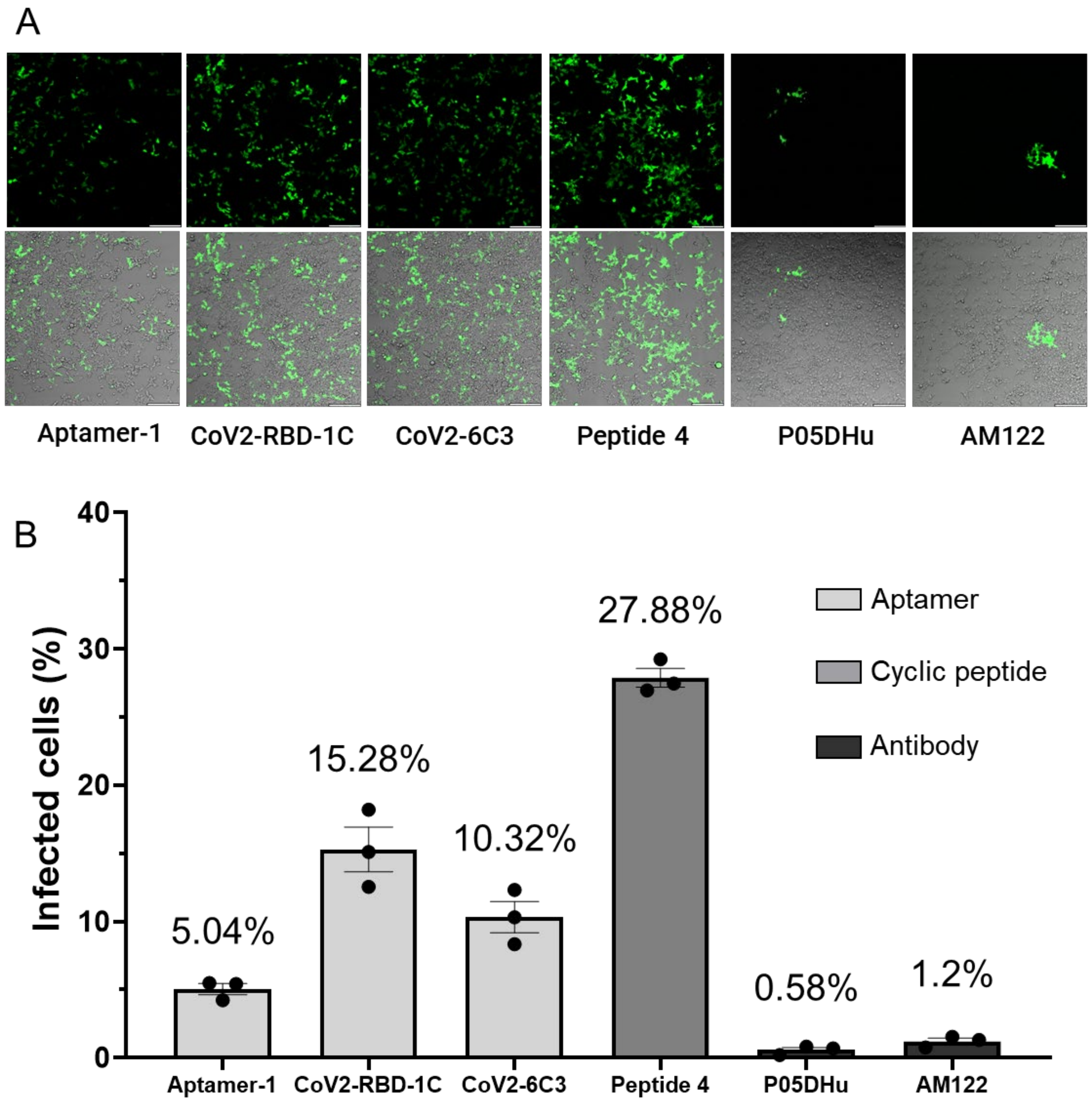

Fig. S10.

Pseudotyped SARS-CoV-2 neutralization assay for the reported RBD binders. (A) Confocal microscopic observation of pseudotyped SARS-CoV-2-infected hACE2-293T cells. The green fluorescence images (top) and the merged images of the green fluorescence with the bright field (bottom) were obtained after viral infection in the presence of the various inhibitors. Scale bars: 200 µm. Excitation: 480 nm and Emission: 530 nm. (**B**) Percentage of GFP-expressing infected cells over the total hACE2-293T cells. Around 5,000 cells were analyzed for each sample. n=3 biological replicates (bars represent means ± s.e.m).

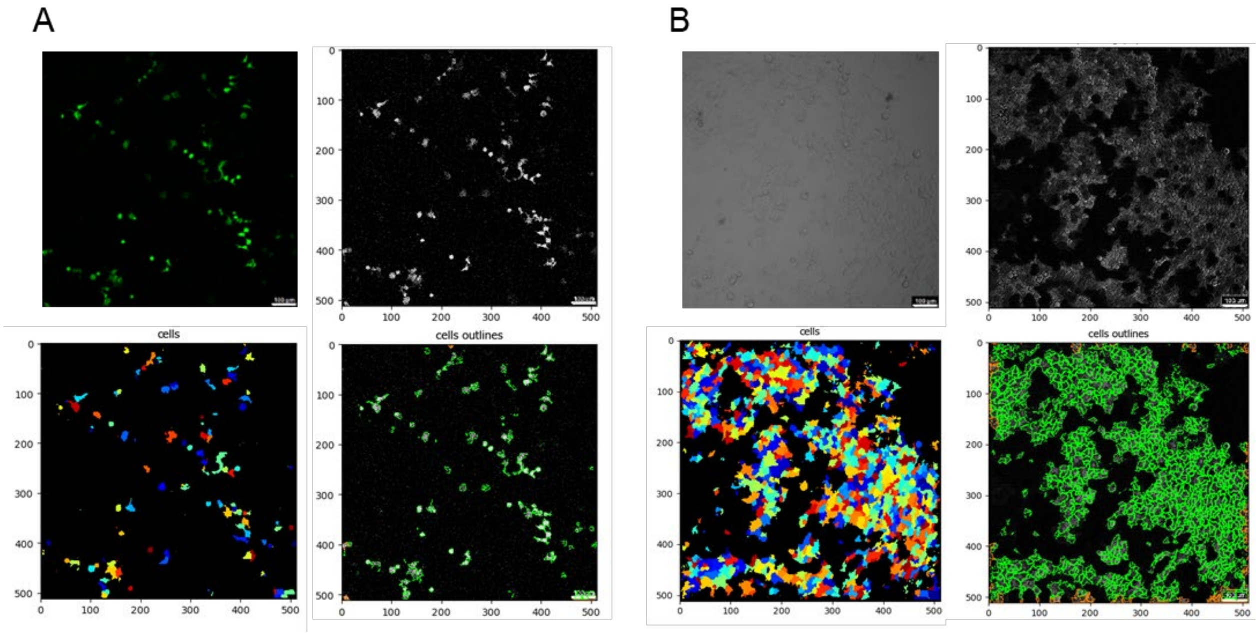

Fig. S11.

Computational analysis of GFP expressing cells. (**A**) The number of pseudotyped SARS-CoV-2-infected hACE2-293T cells was quantitatively analyzed by automatic segmentation of the GFP fluorescence images. (**B**) Automatic segmentation of bright field images represents total number of hACE2-293T.

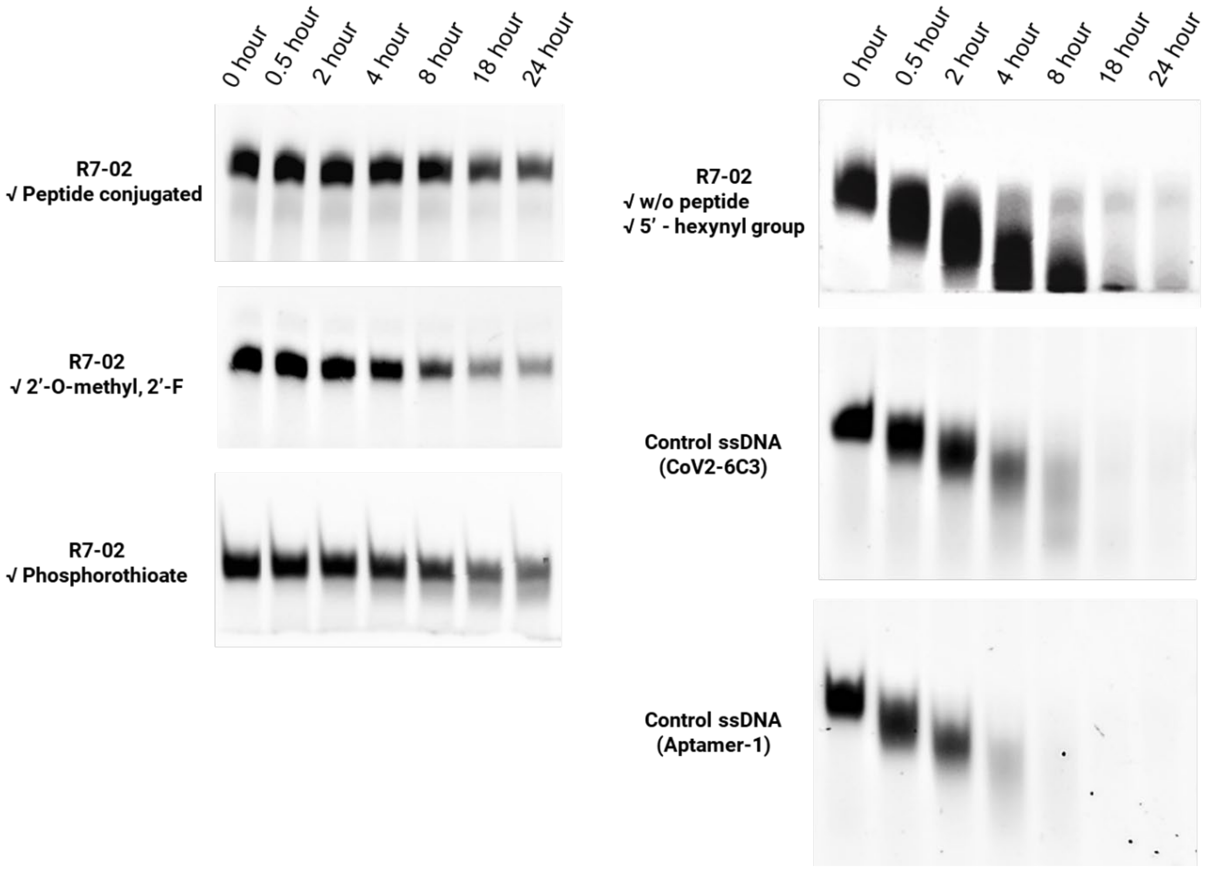

Fig. S12.

Nuclease stability of R7-02. Original and modified R7-02 and the control ssDNA at 1 μM were incubated with 10% FBS in 1xPBS at 37°C for up to 24h. After incubation, samples were heat-denatured in 90°C and analyzed with denaturing 10% polyacrylamide gel electrophoresis.

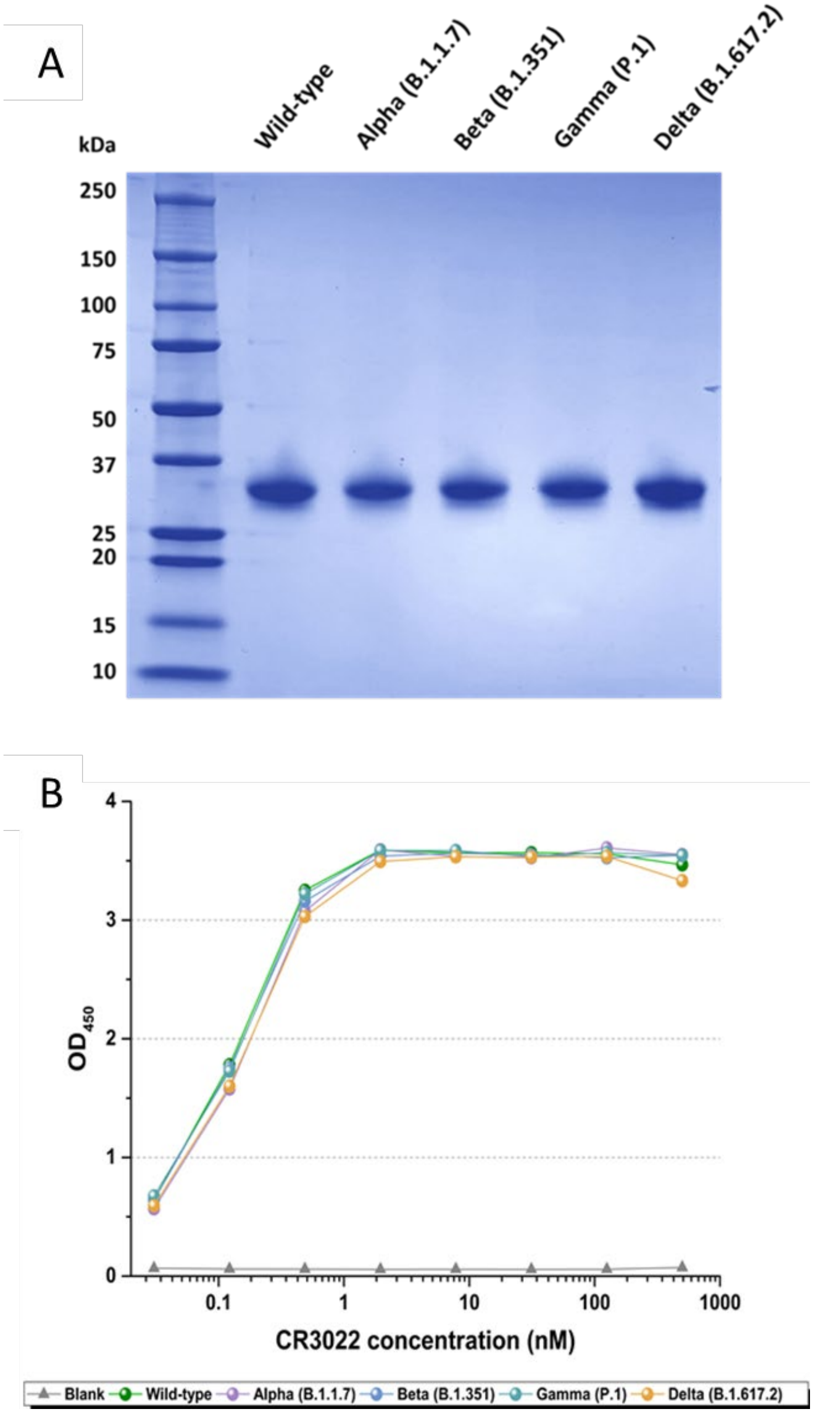

Fig. S13.

Production of SARS-CoV-2 wild-type RBD and its variants in mammalian cells. (**A**) SDS-PAGE analysis showing the purified SARS-CoV-2 RBD proteins (wild-type, Alpha (B.1.1.7), Beta (B.1.351), Gamma (P.1), and Delta (B.1.617.2)). Each RBD protein was expressed in HEK293F cells and purified using Ni-NTA affinity chromatography. (**B**) ELISA results showing the interaction between expressed RBD proteins and CR3022 antibody.

Round	Input hybrid ligand	RBD concentration	Stringency condition
**1**	4ug (200pmol)	200nM	Washing (200 uL, 3 times)
**2**	2ug (100pmol)	100nM	Washing (200 uL, 3 times) + VDC (100x dilution, 20 min)
**3**	2ug (100pmol)	40nM	Washing (200 uL, 3 times) + VDC (500x dilution, 30 min) + Washing (200 uL, 3 times)
**4**	1ug (50pmol)	40nM	Washing (200 uL, 3 times) + VDC (500x dilution, 30 min) + Washing (200 uL, 3 times)
**5**	1ug (50pmol)	40nM	Washing (200 uL, 3 times) + VDC (500x dilution, 30 min) + Washing (200 uL, 3 times)
**6**	1ug (50pmol)	40nM	Washing (200 uL, 3 times) + VDC (500x dilution, 30 min, 2 times) + Washing (200 uL, 3 times)
**7**	1ug (50pmol)	20nM	Washing (200 uL, 3 times) + VDC (500x dilution, 30 min, 2 times) + Washing (200 uL, 3 times)

Table S1.

Selection parameters for each round of HOLD.

ID	Sequence (5' → 3')
**Forward primer**	[Hexynyl]GGAAGAGATGGCGAC
**Reverse primer**	[Phosphate]CCATCAGGATCAGCT
**Random library**	[Hexynyl]GGAAGAGATGGCGAC-N40-AGCTGATCCTGATGG
**(HTS sequencing) forward primer**	AATGATACGGCGACCACCGAGATCTACACTTGGTGAGACACTCTTTCCCTACA CGACGCTCTTCCGATCTNNNNNNNGGAAGAGATGGCGAC
**(HTS sequencing) reverse primer**	CAAGCAGAAGACGGCATACGAGATGTTCAGGTCTGACTGGAGTTCAGACGTGT GCTCTTCCGATCTNNNNNNCCATCAGGATCAGCT
**R7-01**	[Hexynyl]GGAAGAGATGGCGACCTCCACAACCGCTGATCGACGCAGATCTTGTG GGTAGCTGATCCTGATGG[FAM]
**R7-02**	[Hexynyl]GGAAGAGATGGCGACACGCAATCGTGGGTTGGGGATATAATGGTA AGCTGATCCTGATGG[FAM]
**R7-02 (TAMRA)**	[Hexynyl]GGAAGAGATGGCGACACGCAATCGTGGGTTGGGGATATAATGGTA AGCTGATCCTGATGG[TAMRA]
**R7-03**	[Hexynyl]GGAAGAGATGGCGACCACACCAAATCTGAGTCTACCCCATGAAGGA GCTGATCCTGATGG[FAM]
**CoV2-RBD-1C** (*40*)	[TAMRA]CAGCACCGACCTTGTGCTTTGGGAGTGCTGGTCCAAGGGCGTTAATG GACA
**CoV2-6C3** (*41*)	[TAMRA]CGCAGCACCCAAGAACAAGGACTGCTTAGGATTGCGATAGGTTCGG
**Aptamer-1 core** (*12*)	[TAMRA]TCGAGTGGCTTGTTTGTAATGTAGGGTTCCGGTCGTGGGT
**2'-O-Methyl, 2'-F modified R7-02**	[Hexynyl]G(2'-OMe(G))(2'-OMe-(A))(2'-OMe(A))(2'-OMe(G))AGATGGCGACA CGCAATCGTGGGTTGGGGATATAATGGTAAGCTGATCCT(2'-OMe-(G)) (2'-OMe(A))(2'OMe(T))(2'-F(G))G[FAM]
**Phosphorothioate (*) modified R7-02**	[Hexynyl]G*G*A*A*GAGATGGCGACACGCAATCGTGGGTTGGGGATATAATGGT A AGCTGATCCTG*A*T*G*G[FAM]

Table S2.

Oligonucleotides used for HOLD and characterization.

Plasmids	Relevant characteristics	Reference or source
**PMAZ-IGH-GLYCOT**	Trastuzumab H chain gene in PMAZ-IgH-H23	(60)
**PMAZ-SARS-COV-2-WT-RBD-HIS**	SARS-CoV-2 WT-RBD-His gene in pMAZ	Current study
**PMAZ-SARS-COV-2-RBD-ALPHA (B.1.1.7)-HIS**	SARS-CoV-2-RBD-Alpha (B.1.1.7)-His gene in pMAZ	Current study
**PMAZ-SARS-COV-2-RBD-BETA (B.1.351)-HIS**	SARS-CoV-2-RBD-Beta (B.1.351)-His gene in pMAZ	Current study
**PMAZ-SARS-COV-2-RBD-GAMMA (P.1)-HIS**	SARS-CoV-2-RBD-Gamma (P.1)-His gene in pMAZ	Current study
**PMAZ-SARS-COV-2-RBD-DELTA (B.1.617.2)-HIS**	SARS-CoV-2-RBD-Delta (B.1.617.2)-His gene in pMAZ	Current study

Table S3.
List of plasmids used for RBD expression in mammalian cells.

Primer #	Primer sequence (5’ → 3’)
**JS#162**	CGCAGCGAGCGCGCACTCCAGAGTGCAGCCCACCG
**JS#163**	ACACCTCGCCGAAGGGGCACAGATTGGTGATGTTGGGGAACCTCACGATGCTCTCGGTGGGCTGCACTCT
**JS#164**	CCCTTCGGCGAGGTGTTCAACGCCACCAGATTCGCCAGCGTGTATGCTTGGAATAGGAAGAGGATTAGCA
**JS#165**	GCTGAAGCTGGCGCTGTTGTACAGCACGCTGTAGTCGGCCACGCAGTTGCTAATCCTCTTCCTATTCCAA
**JS#166**	CAGCGCCAGCTTCAGCACCTTCAAGTGCTACGGCGTAAGCCCCACCAAGCTGAACGACCTGTGCTTTACC
**JS#167**	ATCTGCCTCACCTCGTCCCCCCTGATCACGAAGCTGTCGGCGTACACATTGGTAAAGCACAGGTCGTTCA
**JS#168**	GGACGAGGTGAGGCAGATCGCTCCCGGTCAGACCGGCAAGATTGCCGATTACAACTACAAGCTGCCTGAC
**JS#169**	TGCTGTCCAGGTTGTTGCTGTTCCAGGCGATCACGCAGCCGGTAAAGTCGTCAGGCAGCTTGTAGTTGTA
**JS#170**	GCAACAACCTGGACAGCAAGGTCGGAGGCAATTACAATTACCTCTACAGACTCTTTCGGAAAAGTAACCT
**JS#171**	TGCCGGCCTGGTAGATCTCTGTGCTGATGTCCCTCTCGAAAGGCTTCAGGTTACTTTTCCGAAAGAGTCT
**JS#172**	GATCTACCAGGCCGGCAGCACCCCCTGTAATGGCGTAGAGGGCTTCAACTGCTACTTCCCCCTGCAGAGC
**JS#173**	ACAACCACCCTGTAGGGCTGGTAGCCAACCCCATTAGTTGGCTGGAAACCGTAGCTCTGCAGGGGGAAGT
**JS#174**	GCCCTACAGGGTGGTTGTACTGAGCTTCGAGCTTCTGCACGCCCCTGCCACCGTGTGCGGCCCTAAGAAA
**JS#175**	AGATCCGTTCTTGACCAAGTTGGTGCTTTTCTTAGGGCCGCACAC
**JS#176**	TTTTAGGGTCTAGATTATCAGTGATGATGGTGGTGATGGTTCTTGACCAAGTTGGTGCTTTTCTTAGG
**JS#179**	GCAGAGCTACGGTTTCCAGCCAACTTATGGGGTTGGCTACCAGCCCTAC
**JS#180**	GTAGGGCTGGTAGCCAACCCCATAAGTTGGCTGGAAACCGTAGCTCTGC
**JS#181**	GCTCCCGGTCAGACCGGCAACATTGCCGATTACAACTACAAGCTGCCTG
**JS#182**	CAGGCAGCTTGTAGTTGTAATCGGCAATGTTGCCGGTCTGACCGGGAGC
**JS#183**	GCAGCACCCCCTGTAATGGCGTAAAGGGCTTCAACTGCTACTTCCCCC
**JS#184**	GGGGGAAGTAGCAGTTGAAGCCCTTTACGCCATTACAGGGGGTGCTGC
**JS#185**	GCTCCCGGTCAGACCGGCACGATTGCCGATTACAACTACAAGCTGCC
**JS#186**	GGCAGCTTGTAGTTGTAATCGGCAATCGTGCCGGTCTGACCGGGAGC
**JS#187**	AGCAAGGTCGGAGGCAATTACAATTACCGCTACAGACTCTTTCGGAAAAGTAACCTGAAGC
**JS#188**	GCTTCAGGTTACTTTTCCGAAAGAGTCTGTAGCGGTAATTGTAATTGCCTCCGACCTTGCT
**JS#191**	GAGATCTACCAGGCCGGCAGCAAACCCTGTAATGGCGTAGAGGGCTTC
**JS#192**	GAAGCCCTCTACGCCATTACAGGGTTTGCTGCCGGCCTGGTAGATCTC

Table S4.

Primers used for RBD expression and purification.

REFERENCES AND NOTES

1. M. S. Maginnis, Virus–receptor interactions: The key to cellular invasion. *J. Mol. Biol.* **430**, 2590–2611 (2018).

2. R. Yan, Y. Zhang, Y. Li, L. Xia, Y. Guo, Q. Zhou, Structural basis for the recognition of SARS-CoV-2 by full-length human ACE2. *Science* **367**, 1444–1448 (2020).

3. D. J. Benton, A. G. Wrobel, P. Xu, C. Roustan, S. R. Martin, P. B. Rosenthal, J. J. Skehel, S. J. Gamblin, Receptor binding and priming of the spike protein of SARS-CoV-2 for membrane fusion. *Nature* **588**, 327–330 (2020).

4. Q. Li, J. Wu, J. Nie, L. Zhang, H. Hao, S. Liu, C. Zhao, Q. Zhang, H. Liu, L. Nie, H. Qin, M. Wang, Q. Lu, X. Li, Q. Sun, J. Liu, L. Zhang, X. Li, W. Huang, Y. Wang, The impact of mutations in SARS-CoV-2 spike on viral infectivity and antigenicity. *Cell* **182**, 1284–1294.e9 (2020).

5. W. T. Harvey, A. M. Carabelli, B. Jackson, R. K. Gupta, E. C. Thomson, E. M. Harrison, C. Ludden, R. Reeve, A. Rambaut; COVID-19 Genomics UK (COG-UK) Consortium, S. J. Peacock, D. L. Robertson, SARS-CoV-2 variants, spike mutations and immune escape. *Nat. Rev. Microbiol.* **19**, 409–424 (2021).

6. J. Li, S. Lai, G. F. Gao, W. Shi, The emergence, genomic diversity and global spread of SARS-CoV-2. *Nature* **600**, 408–418 (2021).

7. X. Zhu, D. Mannar, S. S. Srivastava, A. M. Berezuk, J.-P. Demers, J. W. Saville, K. Leopold, W. Li, D. S. Dimitrov, K. S. Tuttle, S. Zhou, S. Chittori, S. Subramaniam, Cryo-electron microscopy structures of the N501Y SARS-CoV-2 spike protein in complex with ACE2 and 2 potent neutralizing antibodies. *PLOS Biol.* **19**, e3001237 (2021).

8. Y. Liu, J. Liu, K. S. Plante, J. A. Plante, X. Xie, X. Zhang, Z. Ku, Z. An, D. Scharton, C. Schindewolf, S. G. Widen, V. D. Menachery, P.-Y. Shi, S. C. Weaver, The N501Y spike substitution enhances SARS-CoV-2 infection and transmission. *Nature* **602**, 294–299 (2022).

9. D. Corti, L. A. Purcell, G. Snell, D. Veesler, Tackling COVID-19 with neutralizing monoclonal antibodies. *Cell* **184**, 3086–3108 (2021).

10. Y. Xiang, S. Nambulli, Z. Xiao, H. Liu, Z. Sang, W. P. Duprex, D. Schneidman-Duhovny, C. Zhang, Y. Shi, Versatile and multivalent nanobodies efficiently neutralize SARS-CoV-2. *Science* **370**, 1479–1484 (2020).

11. K. K. Chan, D. Dorosky, P. Sharma, S. A. Abbasi, J. M. Dye, D. M. Kranz, A. S. Herbert, E. Procko, Engineering human ACE2 to optimize binding to the spike protein of SARS coronavirus 2. *Science* **369**, 1261–1265 (2020).

12. X. Liu, Y. L. Wang, J. Wu, J. Qi, Z. Zeng, Q. Wan, Z. Chen, P. Manandhar, V. S. Cavener, N. R. Boyle, X. Fu, E. Salazar, S. V. Kuchipudi, V. Kapur, X. Zhang, M. Umetani, M. Sen, R. C. Willson, S. H. Chen, Y. Zu, Neutralizing aptamers block S/RBD-ACE2 interactions and prevent host cell infection. *Angew. Chem. Int. Ed.* **60**, 10273–10278 (2021).

13. P. Wang, M. S. Nair, L. Liu, S. Iketani, Y. Luo, Y. Guo, M. Wang, J. Yu, B. Zhang, P. D. Kwong, B. S. Graham, J. R. Mascola, J. Y. Chang, M. T. Yin, M. Sobieszczyk, C. A. Kyratsous, L. Shapiro, Z. Sheng, Y. Huang, D. D. Ho, Antibody resistance of SARS-CoV-2 variants B.1.351 and B.1.1.7. *Nature* **593**, 130–135 (2021).

14. D. Planas, D. Veyer, A. Baidaliuk, I. Staropoli, F. Guivel-Benhassine, M. M. Rajah, C. Planchais, F. Porrot, N. Robillard, J. Puech, M. Prot, F. Gallais, P. Gantner, A. Velay, J. Le Guen, N. Kassis-Chikhani, D. Edriss, L. Belec, A. Seve, L. Courtellemont, H. Péré, L. Hocqueloux, S. Fafi-Kremer, T. Prazuck, H. Mouquet, T. Bruel, E. Simon-Lorière, F. A. Rey, O. Schwartz, Reduced sensitivity of SARS-CoV-2 variant Delta to antibody neutralization. *Nature* **596**, 276-280 (2021).

15. Y. Cao, J. Wang, F. Jian, T. Xiao, W. Song, A. Yisimayi, W. Huang, Q. Li, P. Wang, R. An, J. Wang, Y. Wang, X. Niu, S. Yang, H. Liang, H. Sun, T. Li, Y. Yu, Q. Cui, S. Liu, X. Yang, S. Du, Z. Zhang, X. Hao, F. Shao, R. Jin, X. Wang, J. Xiao, Y. Wang, X. S. Xie, Omicron escapes the majority of existing SARS-CoV-2 neutralizing antibodies. *Nature* **602**, 657–663 (2022).

16. L. Liu, S. Iketani, Y. Guo, J. F. W. Chan, M. Wang, L. Liu, Y. Luo, H. Chu, Y. Huang, M. S. Nair, J. Yu, K. K. H. Chik, T. T. T. Yuen, C. Yoon, K. K. W. To, H. Chen, M. T. Yin, M. E. Sobieszczyk, Y. Huang, H. H. Wang, Z. Sheng, K.-Y. Yuen, D. D. Ho, Striking antibody evasion manifested by the Omicron variant of SARS-CoV-2. *Nature* **602**, 676–681 (2022).

17. A. Baum, B. O. Fulton, E. Wloga, R. Copin, K. E. Pascal, V. Russo, S. Giordano, K. Lanza, N. Negron, M. Ni, Y. Wei, G. S. Atwal, A. J. Murphy, N. Stahl, G. D. Yancopoulos, C. A. Kyratsous, Antibody cocktail to SARS-CoV-2 spike protein prevents rapid mutational escape seen with individual antibodies. *Science* **369**, 1014-1018 (2020).

18. J. Hansen, A. Baum, K. E. Pascal, V. Russo, S. Giordano, E. Wloga, B. O. Fulton, Y. Yan, K. Koon, K. Patel, K. M. Chung, A. Hermann, E. Ullman, J. Cruz, A. Rafique, T. Huang, J. Fairhurst, C. Libertiny, M. Malbec, W.-Y. Lee, R. Welsh, G. Farr, S. Pennington, D. Deshpande, J. Cheng, A. Watty, P. Bouffard, R. Babb, N. Levenkova, C. Chen, B. Zhang, A. Romero Hernandez, K. Saotome, Y. Zhou, M. Franklin, S. Sivapalasingam, D. C. Lye, S. Weston, J. Logue, R. Haupt, M. Frieman, G. Chen, W. Olson, A. J. Murphy, N. Stahl, G. D. Yancopoulos, C. A. Kyratsous, Studies in humanized mice and convalescent humans yield a SARS-CoV-2 antibody cocktail. *Science* **369**, 1010–1014 (2020).

19. M. McCallum, N. Czudnochowski, L. E. Rosen, S. K. Zepeda, J. E. Bowen, A. C. Walls, K. Hauser, A. Joshi, C. Stewart, J. R. Dillen, A. E. Powell, T. I. Croll, J. Nix, H. W. Virgin, D. Corti, G. Snell, D. Veesler, Structural basis of SARS-CoV-2 Omicron immune evasion and receptor engagement. *Science* **375**, 864–868 (2022).

20. T. N. Starr, N. Czudnochowski, Z. Liu, F. Zatta, Y.-J. Park, A. Addetia, D. Pinto, M. Beltramello, P. Hernandez, A. J. Greaney, R. Marzi, W. G. Glass, I. Zhang, A. S. Dingens, J. E. Bowen, M. A. Tortorici, A. C. Walls, J. A. Wojcechowskyj, A. De Marco, L. E. Rosen, J. Zhou, M. Montiel-Ruiz, H. Kaiser, J. R. Dillen, H. Tucker, J. Bassi, C. Silacci-Fregni, M. P. Housley, J. Di Iulio, G. Lombardo, M. Agostini, N. Sprugasci, K. Culap, S. Jaconi, M. Meury, E. Dellota Jr, R. Abdelnabi, S.-Y. C. Foo, E. Cameroni, S. Stumpf, T. I. Croll, J. C. Nix, C. Havenar-Daughton, L. Piccoli, F. Benigni, J. Neyts, A. Telenti, F. A. Lempp, M. S. Pizzuto, J. D. Chodera, C. M. Hebner, H. W. Virgin, S. P. J. Whelan, D. Veesler, D. Corti, J. D. Bloom, G.

Snell, SARS-CoV-2 RBD antibodies that maximize breadth and resistance to escape. *Nature* **597**, 97–102 (2021).

21. Y.-J. Park, A. De Marco, T. N. Starr, Z. Liu, D. Pinto, A. C. Walls, F. Zatta, S. K. Zepeda, J. E. Bowen, K. R. Sprouse, A. Joshi, M. Giurdanella, B. Guarino, J. Noack, R. Abdelnabi, S.-Y. C. Foo, L. E. Rosen, F. A. Lempp, F. Benigni, G. Snell, J. Neyts, S. P. J. Whelan, H. W. Virgin, J. D. Bloom, D. Corti, M. S. Pizzuto, D. Veesler, Antibody-mediated broad sarbecovirus neutralization through ACE2 molecular mimicry. *Science* **375**, 449–454 (2022).

22. L. Zhang, S. Dutta, S. Xiong, M. Chan, K. K. Chan, T. M. Fan, K. L. Bailey, M. Lindeblad, L. M. Cooper, L. Rong, A. F. Gugliuzza, D. Shukla, E. Procko, J. Rehman, A. B. Malik, Engineered ACE2 decoy mitigates lung injury and death induced by SARS-CoV-2 variants. *Nat. Chem. Biol.* **18**, 342–351 (2022).

23. A. Devi, N. S. N. Chaitanya, Designing of peptide aptamer targeting the receptor-binding domain of spike protein of SARS-CoV-2: An in silico study. *Mol. Divers.* **26**, 157–169 (2022).

24. S. Reverdatto, D. S. Burz, A. Shekhtman, Peptide aptamers: Development and applications. *Curr. Top. Med. Chem.* **15**, 1082–1101 (2015).

25. S. Kim, D. Kim, Y. Lee, H. Jeon, B.-H. Lee, S. Jon, Conversion of low-affinity peptides to high-affinity peptide binders by using a β-hairpin scaffold-assisted approach. *Chembiochem* **16**, 43–46 (2015).

26. T. A. Knappe, F. Manzenrieder, C. Mas-Moruno, U. Linne, F. Sasse, H. Kessler, X. Xie, M. A. Marahiel, Introducing lasso peptides as molecular scaffolds for drug design: Engineering of an integrin antagonist. *Angew. Chem. Int. Ed.* **50**, 8714–8717 (2011).

27. M. A. Dechantsreiter, E. Planker, B. Mathä, E. Lohof, G. Hölzemann, A. Jonczyk, S. L. Goodman, H. Kessler, *N*-methylated cyclic RGD peptides as highly active and selective $\alpha_V\beta_3$ integrin antagonists. *J. Med. Chem.* **42**, 3033–3040 (1999).

28. Y. Han, P. Kral, Computational design of ACE2-based peptide inhibitors of SARS-CoV-2. *ACS Nano* **14**, 5143–5147 (2020).

29. P. Karoyan, V. Vieillard, L. Gomez-Morales, E. Odile, A. Guihot, C. E. Luyt, A. Denis, P. Grondin, O. Lequin, Human ACE2 peptide-mimics block SARS-CoV-2 pulmonary cells infection. *Commun. Biol.* **4**, 197 (2021).

30. J. Yang, S. J. L. Petitjean, M. Koehler, Q. Zhang, A. C. Dumitru, W. Chen, S. Derclaye, S. P. Vincent, P. Soumillion, D. Alsteens, Molecular interaction and inhibition of SARS-CoV-2 binding to the ACE2 receptor. *Nat. Commun.* **11**, 4541 (2020).

31. A. D. Ellington, J. W. Szostak, Selection in vitro of single-stranded DNA molecules that fold into specific ligand-binding structures. *Nature* **355**, 850–852 (1992).

32. K. T. Savjani, A. K. Gajjar, J. K. Savjani, Drug solubility: Importance and enhancement techniques. *ISRN Pharm.* **2012**, 1–10 (2012).

33. J. A. Kulkarni, D. Witzigmann, S. B. Thomson, S. Chen, B. R. Leavitt, P. R. Cullis, R. Van Der Meel, The current landscape of nucleic acid therapeutics. *Nat. Nanotechnol.* **16**, 630–643 (2021).

34. S. Kang, S. S. Hah, Improved ligand binding by antibody-aptamer pincers. *Bioconjug. Chem.* **25**, 1421–1427 (2014).

35. H. Hasegawa, N. Savory, K. Abe, K. Ikebukuro, Methods for improving aptamer binding affinity. *Molecules* **21**, 421 (2016).

36. J. Shang, G. Ye, K. Shi, Y. Wan, C. Luo, H. Aihara, Q. Geng, A. Auerbach, F. Li, Structural basis of receptor recognition by SARS-CoV-2. *Nature* **581**, 221–224 (2020).

37. M. Kimoto, R. Yamashige, K. Matsunaga, S. Yokoyama, I. Hirao, Generation of high-affinity DNA aptamers using an expanded genetic alphabet. *Nat. Biotechnol.* **31**, 453–457 (2013).

38. J. D. Vaught, C. Bock, J. Carter, T. Fitzwater, M. Otis, D. Schneider, J. Rolando, S. Waugh, S. K. Wilcox, B. E. Eaton, Expanding the chemistry of DNA for in vitro selection. *J. Am. Chem. Soc.* **132**, 4141–4151 (2010).

39. T. Kanagawa, Bias and artifacts in multitemplate polymerase chain reactions (PCR). *J. Biosci. Bioeng.* **96**, 317–323 (2003).

40. Y. Song, J. Song, X. Wei, M. Huang, M. Sun, L. Zhu, B. Lin, H. Shen, Z. Zhu, C. Yang, Discovery of aptamers targeting the receptor-binding domain of the SARS-CoV-2 spike glycoprotein. *Anal. Chem.* **92**, 9895–9900 (2020).

41. M. Sun, S. Liu, X. Wei, S. Wan, M. Huang, T. Song, Y. Lu, X. Weng, Z. Lin, H. Chen, Y. Song, C. Yang, Aptamer blocking strategy inhibits SARS-CoV-2 virus infection. *Angew. Chem. Int. Ed.* **60**, 10266–10272 (2021).

42. A. Norman, C. Franck, M. Christie, P. M. E. Hawkins, K. Patel, A. S. Ashhurst, A. Aggarwal, J. K. K. Low, R. Siddiquee, C. L. Ashley, M. Steain, J. A. Triccas, S. Turville, J. P. Mackay, T. Passioura, R. J. Payne, Discovery of cyclic peptide ligands to the SARS-CoV-2 spike protein using mRNA display. *ACS Cent. Sci.* **7**, 1001–1008 (2021).

43. L. Piccoli, Y.-J. Park, M. A. Tortorici, N. Czudnochowski, A. C. Walls, M. Beltramello, C. Silacci-Fregni, D. Pinto, L. E. Rosen, J. E. Bowen, O. J. Acton, S. Jaconi, B. Guarino, A. Minola, F. Zatta, N. Sprugasci, J. Bassi, A. Peter, A. De Marco, J. C. Nix, F. Mele, S. Jovic, B. F. Rodriguez, S. V. Gupta, F. Jin, G. Piumatti, G. Lo Presti, A. F. Pellanda, M. Biggiogero, M. Tarkowski, M. S. Pizzuto, E. Cameroni, C. Havenar-Daughton, M. Smithey, D. Hong, V. Lepori, E. Albanese, A. Ceschi, E. Bernasconi, L. Elzi, P. Ferrari, C. Garzoni, A. Riva, G. Snell, F. Sallusto, K. Fink, H. W. Virgin, A. Lanzavecchia, D. Corti, D. Veesler, Mapping neutralizing and immunodominant sites on the SARS-CoV-2 spike receptor-binding domain by structure-guided high-resolution serology. *Cell* **183**, 1024–1042 (2020).

44. J. Nie, Q. Li, J. Wu, C. Zhao, H. Hao, H. Liu, L. Zhang, L. Nie, H. Qin, M. Wang, Q. Lu, X. Li, Q. Sun, J. Liu, C. Fan, W. Huang, M. Xu, Y. Wang, Quantification of SARS-CoV-2 neutralizing antibody by a pseudotyped virus-based assay. *Nat. Protoc.* **15**, 3699–3715 (2020).

45. Z. Ke, J. Oton, K. Qu, M. Cortese, V. Zila, L. McKeane, T. Nakane, J. Zivanov, C. J. Neufeldt, B. Cerikan, J. M. Lu, J. Peukes, X. Xiong, H.-G. Kräusslich, S. H. W. Scheres, R.

Bartenschlager, J. A. G. Briggs, Structures and distributions of SARS-CoV-2 spike proteins on intact virions. *Nature* **588**, 498-502 (2020).

46. M. Han, J. Beon, J. Y. Lee, S. S. Oh, Systematic combination of oligonucleotides and synthetic polymers for advanced therapeutic applications. *Macromol. Res.* **29**, 665–680 (2021).

47. M. Muttenthaler, G. F. King, D. J. Adams, P. F. Alewood, Trends in peptide drug discovery. *Nat. Rev. Drug Discov.* **20**, 309–325 (2021).

48. N. Nakatsuka, K.-A. Yang, J. M. Abendroth, K. M. Cheung, X. Xu, H. Yang, C. Zhao, B. Zhu, Y. S. Rim, Y. Yang, P. S. Weiss, M. N. Stojanović, A. M. Andrews, Aptamer-field-effect transistors overcome Debye length limitations for small-molecule sensing. *Science* **362**, 319–324 (2018).

49. B. Kang, S. V. Park, H. T. Soh, S. S. Oh, A dual-sensing DNA nanostructure with an ultrabroad detection range. *ACS Sens.* **4**, 2802–2808 (2019).

50. H. Yoo, J. Y. Lee, K. S. Park, S. S. Oh, Lead-start isothermal polymerase amplification controlled by DNAzymatic switches. *Nanoscale* **14**, 7828–7836 (2022).

51. R. Ueki, S. Uchida, N. Kanda, N. Yamada, A. Ueki, M. Akiyama, K. Toh, H. Cabral, S. Sando, A chemically unmodified agonistic DNA with growth factor functionality for in vivo therapeutic application. *Sci. Adv.* **6**, eaay2801 (2020).

52. F. Sutanto, S. Shaabani, R. Oerlemans, D. Eris, P. Patil, M. Hadian, M. Wang, M. E. Sharpe, M. R. Groves, A. Dömling, Combining high-throughput synthesis and high-throughput protein crystallography for accelerated hit identification. *Angew. Chem. Int. Ed.* **60**, 18231–18239 (2021).

53. J.-P. Renaud, A. Chari, C. Ciferri, W.-t. Liu, H.-W. Rémigy, H. Stark, C. Wiesmann, Cryo-EM in drug discovery: Achievements, limitations and prospects. *Nat. Rev. Drug Discov.* **17**, 471–492 (2018).

54. H. Yoo, H. Jo, S. S. Oh, Detection and beyond: Challenges and advances in aptamer-based biosensors. *Mater. Adv.* **1**, 2663–2687 (2020).

55. S. V. Park, J.-S. Yang, H. Jo, B. Kang, S. S. Oh, G. Y. Jung, Catalytic RNA, ribozyme, and its applications in synthetic biology. *Biotechnol. Adv.* **37**, 107452 (2019).

56. B. Townshend, J. S. Xiang, G. Manzanarez, E. J. Hayden, C. D. Smolke, A multiplexed, automated evolution pipeline enables scalable discovery and characterization of biosensors. *Nat. Commun.* **12**, 1437 (2021).

57. A. Bashir, Q. Yang, J. Wang, S. Hoyer, W. Chou, C. McLean, G. Davis, Q. Gong, Z. Armstrong, J. Jang, H. Kang, A. Pawlosky, A. Scott, G. E. Dahl, M. Berndl, M. Dimon, B. S. Ferguson, Machine learning guided aptamer refinement and discovery. *Nat. Commun.* **12**, 2366 (2021).

58. M. Cho, S. S. Oh, J. Nie, R. Stewart, M. Eisenstein, J. Chambers, J. D. Marth, F. Walker, J. A. Thomson, H. T. Soh, Quantitative selection and parallel characterization of aptamers. *Proc. Natl. Acad. Sci. U.S.A.* **110**, 18460–18465 (2013).

59. S. S. Oh, K. M. Ahmad, M. Cho, S. Kim, Y. Xiao, H. T. Soh, Improving aptamer selection efficiency through volume dilution, magnetic concentration, and continuous washing in microfluidic channels. *Anal. Chem.* **83**, 6883–6889 (2011).

60. S. T. Jung, S. T. Reddy, T. H. Kang, M. J. Borrok, I. Sandlie, P. W. Tucker, G. Georgiou, Aglycosylated IgG variants expressed in bacteria that selectively bind FcγRI potentiate tumor cell killing by monocyte-dendritic cells. *Proc. Natl. Acad. Sci. U.S.A.* **107**, 604-609 (2010).
